# Iron Induces Resistance Against the Rice Blast Fungus *Magnaporthe oryzae* Through Potentiation of Immune Responses

**DOI:** 10.1186/s12284-022-00609-w

**Published:** 2022-12-25

**Authors:** Ferran Sánchez-Sanuy, Roberto Mateluna-Cuadra, Keisuke Tomita, Kazunori Okada, Gian Attilio Sacchi, Sonia Campo, Blanca San Segundo

**Affiliations:** 1 Centre for Research in Agricultural Genomics (CRAG), CSIC-IRTA-UAB-UB, Campus Universitat Autónoma de Barcelona (UAB), Bellaterra (Cerdanyola del Vallés), Barcelona, Spain; 2 Biotechnology Research Center, The University of Tokyo, Tokyo, Japan; 3 Dipartimento di Scienze Agrarie e Ambientali, Università degli Studi di Milano, Milan, Italy; 4 Consejo Superior de Investigaciones Científicas (CSIC), Barcelona, Spain; 5 Present Address: Fundació Miquel Agustí, Campus Baix Llobregat, Castelldefels, Barcelona, Spain

**Keywords:** Blast, Ferroptosis, Immune response, Iron, *Magnaporthe oryzae*, *Oryza sativa*, Phytoalexins

## Abstract

Iron is an essential nutrient required for plant growth and development. The availability of iron might also influence disease resistance in plants. However, the molecular mechanisms involved in the plant response to iron availability and immunity have been investigated separately from each other. In this work, we found that exposure of rice plants to high iron enhances resistance to infection by the fungal pathogen *Magnaporthe oryzae*, the causal agent of blast disease. RNA-Seq analysis revealed that blast resistance in iron-treated rice plants was associated with superinduction of defense-related genes during pathogen infection, including *Pathogenesis-Related* genes. The expression level of genes involved in the biosynthesis of phytoalexins, both diterpene phytoalexins and the flavonoid phytoalexin sakuranetin, was also higher in iron-treated plants compared with control plants, which correlated well with increased levels of phytoalexins in these plants during *M. oryzae* infection. Upon pathogen infection, lipid peroxidation was also higher in iron-treated plants compared with non-treated plants. We also show that *M. oryzae* infection modulates the expression of genes that play a pivotal role in the maintenance of iron homeostasis. Histochemical analysis of *M. oryzae*-infected leaves revealed colocalization of iron and reactive oxygen species in cells located in the vicinity of fungal penetration sites (e.g. appressoria) in rice plants that have been exposed to iron. Together these findings support that ferroptosis plays a role in the response of iron-treated rice plants to infection by virulent *M. oryzae.* Understanding interconnected regulations between iron signaling and immune signaling in rice holds great potential for developing novel strategies to improve blast resistance in rice.

**Supplementary Information:**

The online version contains supplementary material available at 10.1186/s12284-022-00609-w.

## Background

Iron (Fe) is an essential nutrient element required for important processes in plant growth and development, such as photosynthesis and mitochondrial respiration. It is commonly associated to cellular reduction–oxidation (redox) reactions and electron transfer chains (Connorton et al. 2017). Although iron is abundant in most agricultural soils, its bioavailability to plants is usually very low. In alkaline soils, Fe is predominantly found in the form of insoluble ferric form (Fe^3+^), forming hydroxide complexes poorly available for the plant. In contrast, under reductive conditions, Fe is present as the soluble ferrous form (Fe^2+^). Both deficiency and excess of Fe can be toxic to the plant. In particular, excess Fe leads to the generation of reactive oxygen species (ROS) through redox reactions between Fe^3+^ and Fe^2+^ (Haber–Weiss and Fenton reactions) which can be harmful to the plant. ROS can damage lipids (e.g. membrane lipids), proteins and nucleic acids and eventually, cell death (Mansoor et al. 2022). Therefore, plants have evolved homeostatic mechanisms to regulate Fe accumulation and provide enough amounts for the plant metabolism while preventing Fe accumulation causing oxidative stress.

Plants use two main approaches to take up Fe from the soil, referred to as Strategy I and Strategy II (also known as reducing and chelating strategies, respectively) (Marschner and Römheld 1994). The main difference between both strategies is the oxidation state of Fe when taken up by the plant, ferrous Fe^2+^ for Strategy I and ferric Fe^3+^ for Strategy II. The strategy I (reduction strategy) is found in all plants except those from the *Poaceae* family, and has been characterized in the model plant *Arabidopsis thaliana*. It consists in lowering soil pH by extrusion of protons to increase Fe solubility which relies on plasma membrane H^+^-ATPases (Santi and Schmidt 2009). The Fe^3+^ is then reduced to Fe^2+^ by a ferric reductase-oxidases (FROs) located in the plasma membrane and imported into the root epidermal cells by iron-regulated transporters (IRTs) (Eide et al. 1996; Robinson et at. 1999). Additional mechanisms used in Strategy I plants involves the release of Fe chelating compounds, such as phenolic compounds, to the rhizosphere (Rodríguez-Celma et al. 2013). In Strategy II (chelation strategy), which is found in graminaceous species, the roots produce phytosiderophores (PS) which are transported to the rhizosphere by TOM (Transporter of mugineic acid) that chelate Fe^3+^ in the soil (Nozoye et al. 2011). The PS-Fe^3+^ complexes are then transported into the root cells by yellow stripe-like (YSL) transporters (Nozoye et al. 2011). Rice, unlike most graminaceous crops, is well adapted for growth under submerged conditions (paddy fields) where Fe^2+^ is frequently more abundant than Fe^3+^. Under such conditions, the absorption of excessive Fe^2+^ by the rice roots might cause oxidative stress and toxicity. Rice, in spite of being a grass, uses a combined strategy for Fe uptake which has features of Strategy II (PS release through TOM and PS-Fe^3+^ uptake through YSL), as well as features of Strategy I (Fe^2+^ uptake using IRT transporters) (Ishimaru et al. 2006; Inoue et al. 2009; Nozoye et al. 2011).

Due to its potential toxicity, plants develop mechanisms to regulate the transport, utilization and storage of Fe. For translocation, Fe is complexed to chelators, namely the phytosiderophore deoxymugineic acid (DMA). In the xylem, Fe^3+^-citrate is the dominant form of Fe transport (Connorton et al. 2017). Fe storage mechanisms in plants include sequestration into vacuoles as well as binding to Ferritins (Aung and Masuda 2020). Compared to roots, our knowledge on mechanisms of regulation of Fe transport and homeostasis in leaf tissues is still limited.

The iron nutritional status might have an effect on disease resistance in plants (Herlihy et al. 2020; Liu et al. 2007). Considering that foliar pathogens entirely depend on the host tissue to acquire this vital element, during pathogen infection there is a competition between the host and the pathogen for Fe. In plant/pathogen interactions, the plant might use different strategies to reduce pathogen virulence. On the one side, plants might use iron-withholding strategies by sequestering iron from pathogens, a phenomenon originally described in animals and referred to as “nutritional immunity” (Weinberg 1975). On the other hand, the host plant might capitalize the toxicity of iron through local accumulation of Fe that can be toxic for the invading pathogen (Herlihy et al. 2020). Plant pathogens might also deploy strategies for Fe acquisition from the host plant, such as the secretion of high-affinity Fe-binding siderophores (Herlihy et al. 2020). The deployment of these strategies by the host and/or the pathogen might determine the outcome of the interaction.

Sensing of Fe depletion has been proposed as a mechanism by which plants recognize a pathogen threat for the activation of immune responses (Herlihy et al. 2020). Supporting this notion, resistance to infection by *Dickeya dadantii* and *Botrytis cinerea* was observed in Fe-starved *Arabidopsis* plants (Kieu et al. 2012). In other studies, Fe-starved maize plants were found to be unable to produce ROS during *Colletotrichum graminicola* infection which correlated with increased susceptibility to this fungal pathogen (Ye et al. 2014). On the other hand, local excess iron can activate ROS burst for pathogen resistance as recently described in maize plants during infection by *Curvularia lunata* causing leaf spot (Fu et al. 2022). At present, crosstalk mechanisms that adjust iron homeostasis and immune responses in plants are not fully understood, particularly in crop species.

Rice (*Oryza sativa* L.) is one of the most important crops in the world, and its production is severely affected by the fungus *Magnaporthe oryzae*. This fungus is the causal agent of the rice blast disease (Wilson and Talbot 2009; Fernandez and Orth 2018). In previous studies, it was described that Fe^3+^ and ROS (e.g. H_2_O_2_) accumulate in rice leaves during infection with an avirulent strain of *M. oryzae* (Dangol et al. 2019). In this work, we investigated the effect of treatment with high Fe on blast resistance, at the molecular and cellular level. RNA-Seq analysis revealed that short exposure of rice plants with high Fe is accompanied by stronger induction of defense responses, including *Pathogenesis-Related* (*PR*) gene expression. Phytoalexin biosynthesis genes were also strongly induced by *M. oryzae* infection in Fe-treated rice plants, which correlates well with a higher accumulation of phytoalexins in these plants. Superactivation of defense genes is associated to resistance to *M. oryzae* infection in Fe-treated rice plants. Pathogen infection also modulates the expression of genes involved in iron homeostasis in rice leaves and provokes alterations in Fe content (total Fe, apoplastic Fe). Histochemical staining revealed co-localization in ROS and Fe during infection of Fe-treated rice plants with a virulent *M. oryzae* isolate (compatible interaction), these plants also showing increased levels of lipid peroxidation during *M. oryzae* infection. Treatment with the ferroptosis inhibitor Ferrostatin-1 (Fer-1) suppresses Fe accumulation suggesting that ferroptosis underlies blast resistance in Fe-treated rice plants. Collectively, these results further support a relationship between the Fe signaling and immune signaling in rice while highlighting the relevance of ferroptosis in conferring blast resistance in rice.

## Results

### Treatment with High Fe Enhances Resistance to *M. oryzae* Infection in Rice Plants

**Fig. 1:**
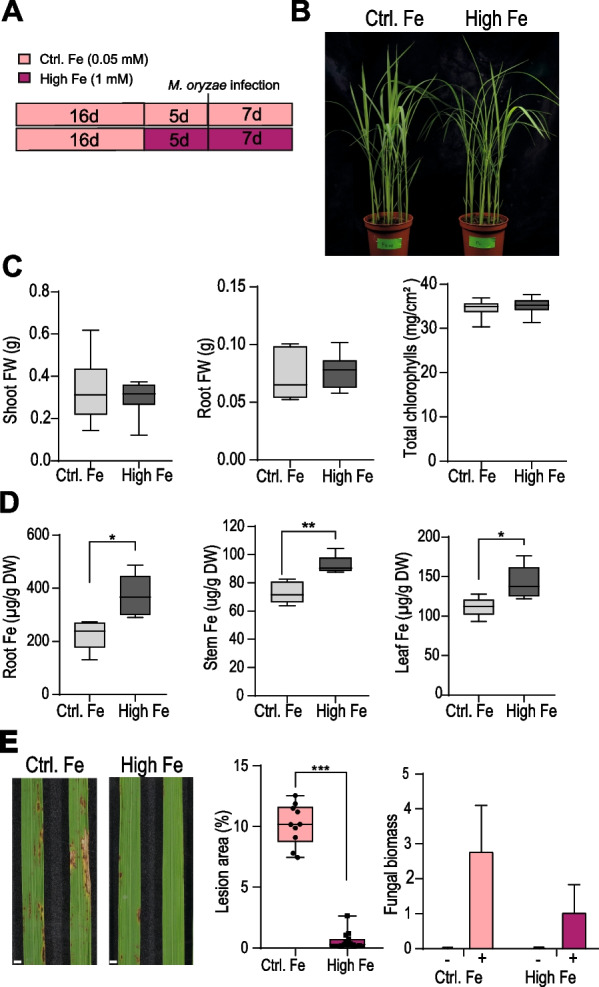
Resistance to *M. oryzae* infection in rice plants grown under high Fe supply. **A** Experimental design used for iron treatment and infection with *M. oryzae* (5 × 10^5^ spores/ml). Rice plants were grown in soil for 16 days under sufficient Fe supply (50 µM Fe, Control plants) and allowed to continue growth under sufficient conditions (Ctrl) or high Fe conditions (1 mM Fe; High Fe) for 5 days more (left panel). Then, the rice plants were inoculated or not with *M. oryzae* spores and assayed for blast resistance. **B** Appearance of rice plants that have been treated with high Fe for 5 days. **C** Shoot and root fresh weight (FW, left and middle panels), and chlorophyll content (right panel) of plants treated with high Fe for 5 days. Differences were not statistically significant. **D** Total Fe content estimated by the ferrozine colorimetric method in roots, stems and leaves of Control and High Fe-treated plants (5 days of treatment). Data are mean ± SEM (n = 10). Asterisks indicate statistical significance calculated by *t*-test (*, *P* < 0.05; **, *P* < 0.01). **E** Resistance to *M. oryzae* in Fe-treated rice plants. Control and High Fe-treated rice plants were inoculated with a conidial suspension of *M. oryzae* spores (5 × 10^5^ spores/ml), or mock-inoculated. Left panel, Disease symptoms in youngest developed leaves (3rd leaf) at 7 days post-inoculation (dpi) are shown. Results from one representative experiment of four independent experiments are presented. Middle panel, percentage of diseased area at 7 dpi (n = 10). Right panel, fungal biomass was quantified by qPCR using specific primers of the *M. oryzae 28S* ribosomal gene relative to the rice *Ubiquitin 1* gene (Os06g46770) at 7 dpi, (−, mock-inoculated; +, *M. oryzae*-inoculated). Data are mean ± SEM (n = 10). Asterisks indicate statistically significant differences calculated by *t*-test (***, *P* < 0.001)

In this work, we investigated the effect of treatment with high Fe on resistance to infection by *M. oryzae* in rice plants. Rice (*O. sativa* cv. Nipponbare) plants were grown in soil under sufficient Fe supply (50 µM Fe) for 16 days. Then, half of the plants were supplied with 1 mM Fe while the other half of the plants continued growth under 50 µM Fe condition (henceforth High-Fe and Control Fe plants, respectively). Treatment with high Fe was carried out for 5 days (Fig. 1A). No phenotypic differences were observed between Control and High-Fe plants after 5 days of Fe treatment (Fig. 1B), these plants showing similar shoot biomass, root biomass and chlorophyll content (Fig. 1C). High-Fe plants had a higher content of Fe in root, stem and leaf tissues compared with Control Fe plants (Fig. 1D). Under the same experimental conditions, cupper, manganese or calcium levels were not significantly altered in High-Fe plants compared with Control-Fe plants (Additional file 1: Fig. S1A). When using a longer period of Fe treatment (e.g. 19 days of treatment with 1 mM Fe), plants developed symptoms of Fe toxicity in their leaves, these plants also showing a significant reduction in root and shoot biomass and chlorophyll content (Additional file 1: Fig. S1B, C). To avoid that toxic effects caused by Fe accumulation could confound our results on disease resistance, in this study we used a short-term Fe treatment (5 days of treatment with 1 mM Fe), followed by a *M. oryzae* infection for 7 days (Fig. 1A).

Most importantly, upon infection with *M. oryzae*, High-Fe plants consistently exhibited resistance to infection as revealed by visual inspection of disease symptoms, quantification of diseased leaf area and measurement of fungal biomass in leaves of pathogen-infected plants (Fig. 1E). These results were substantially similar to those previous reported in hydroponically-grown rice plants (Peris-Peris et al. 2017) supporting that treatment with high Fe increases resistance to infection by the rice blast fungus in rice.

### Transcriptional Responses of Rice Leaves to Treatment with High Fe

Transcriptional changes occurring in rice leaves in response to treatment with high Fe were examined. For this, RNA-Seq analysis was carried out in leaves from High-Fe and Control plants. Differentially expressed genes (DEGs) were identified based on significance level (FDR ≤ 0.05) and log_2_ fold change (FC) with a threshold of FC ≥  + 0.5 and FC ≤  − 0.5 for up-regulated and down-regulated genes, respectively.

**Fig. 2:**
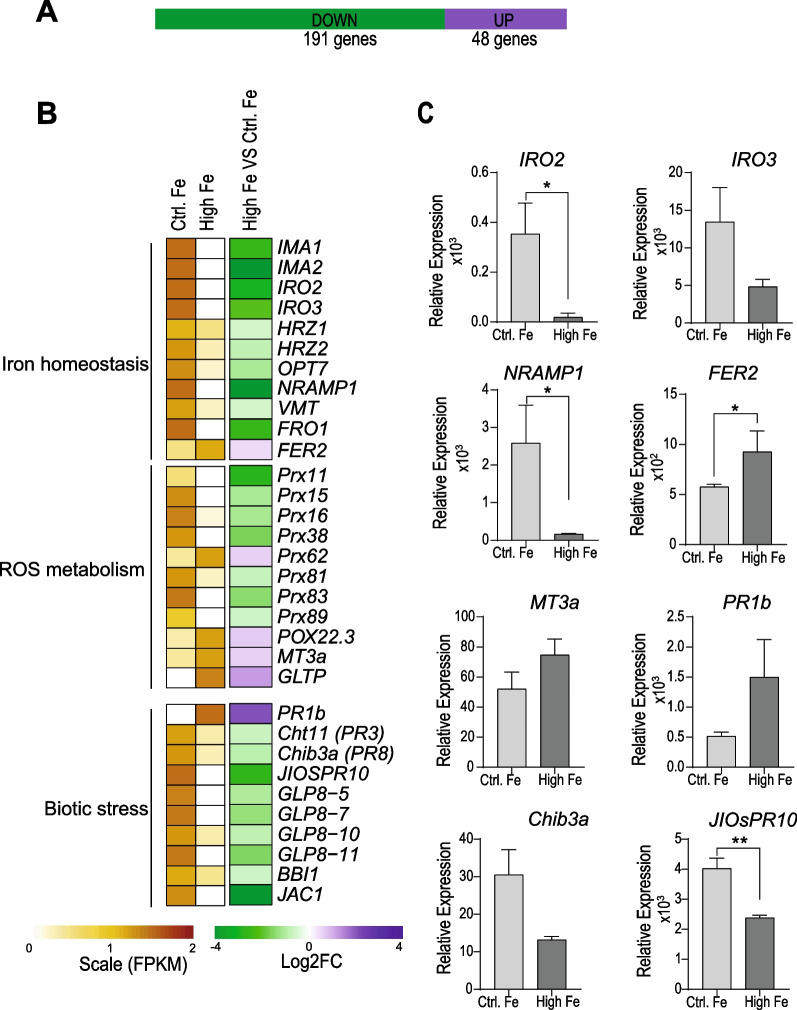
Differentially expressed genes in leaves of High-Fe plants relative to Control plants.** A** Number of genes that are up-regulated and down-regulated genes in leaves of rice plants treated with high Fe for 5 days.** B** Heatmap showing the expression of genes involved in Fe homeostasis, ROS metabolism and biotic stress identified as differentially expressed by RNA-Seq analysis. The expression level (row scaled FPKM, fragments per kilobase per million reads) is represented from pale yellow (less expressed) to brown (more expressed). Fold change (FC) values are represented on the right, in purple (up-regulated genes, Log_2_ FC ≥  + 0.5) and green (down-regulated genes, Log_2_ FC ≥  − 0.5). Data represented correspond to the mean of two biological replicates, each biological replicate consisting in a pool of 5 leaves from individual plants. The full gene name and ID of DEGs are indicated in Additional file 2: Table S1. **C** RT-qPCR analysis of DEGs in High-Fe plants relative to Control plants. Data are mean ± SEM (n = 4 biological replicates, including the two replicates used for RNA-Seq analysis). Asterisks indicate statistically significant differences (*t*-test, *, *P* < 0.05; **, *P* < 0.01). Gene-specific primers are listed in Additional file 9: Table S8

When comparing leaf transcriptomes of High-Fe and Control plants, only 239 genes were found to be regulated by treatment with high Fe (48 genes upregulated; 191 genes down-regulated) (Fig. 2A, Additional file 2: Table S1a). Genes whose expression is known to be induced by Fe deficiency where found to be repressed in leaves of High-Fe plants relative to Control plants (Fig. 2B, Additional file 2: Table S1b) which is consistent with an increase in Fe content in these plants (see Fig. 1C). Down-regulated genes in High-Fe plants included: *Iron deficiency-inducible peptide-IRON MAN* (*OsIMA1*, *OsIMA2*), *Iron-related transcription factors* (*OsIRO2, OsIRO3*) and *Iron-binding Haemerythrin RING ubiquitin ligases* (*OsHRZ1, OsHRZ2*). Other genes down-regulated in leaves of High-Fe rice plants were those encoding plasma membrane iron transporters, such as *Iron-deficiency-regulated oligopeptide transporter 7* (*OsOPT7*), *Natural Resistance-Associated Macrophage Protein 1* (*OsNRAMP1*), Vacuolar Membrane Ferric-Chelate Reductase (*OsFRO1*), and Vacuolar mugineic acid transporter (*OsVMT*) (Fig. 2B, Additional file 2: Table S1a). *OsFER2* (*FERRITIN 2*) was up-regulated in leaves of High-Fe plants (Fig. 2B). As previously mentioned, Ferritins bind Fe (mainly in the form of Fe^3+^), thus, contributing to protection of plants against Fe-mediated oxidative stress.

Transcriptome analysis also indicated that treatment with high Fe results in alterations in the expression of genes involved in ROS metabolism, in particular peroxidase (*prx*) genes (Fig. 2B, Additional file 2: Table S1a). Compared with Control plants, the expression of *prx* genes was either up-regulated (*OsPrx62, OsPOX22.3*) or down-regulated (*OsPrx11, OsPrx15, OsPrx16, OsPrx38, OsPrx81, OsPrx83, OsPrx89*) in High-Fe plants. Peroxidases participate in a broad range of processes, such as cross-linking of cell wall components, synthesis of phytoalexins, and ROS production in plant defense responses. They catalyze oxidation of various substrates concomitant with the decomposition of H_2_O_2_. Among peroxidases, Class III peroxidases can either produce H_2_O_2_ in oxidase cycle or scavenge H_2_O_2_ to H_2_O and O_2_ through peroxidase activity. POX22.3 was previously identified among proteins accumulating in *M. oryzae*-infected rice leaves (Sun et al. 2004). A *Metallothionein-like protein* (*OsMT3a*), and a glycolipid transfer protein involved in ceramide transport (*OsGLTP*) were also up-regulated in High-Fe plants compared with Control plants (Fig. 2B). Metallothioneins are metal-binding proteins implicated in metal detoxification and scavenging of ROS (Hassinen et al. 2011), while ceramides regulate the Arabidopsis defense response by promoting H_2_O_2_ generation (Li et al. 2022).

Genes related to responses to biotic stress were also regulated by Fe treatment under non-infection conditions, including certain *Pathogenesis-Related* (*PR*) genes. Their expression was up-regulated (*OsPR1b*) or down-regulated (*OsCht11* and *OsChib3a* encoding rice chitinases; *Jasmonate-Inducible PR10,* or *JIOsPR10*; and *Germin-like Protein,* or *OsGLP* genes) in High-Fe plants (Fig. 2B).

Results obtained by RNA-Seq analysis were validated by RT-qPCR for genes showing either down-regulation (*OsIRO2, OsIRO3, OsNRAMP1, OsChib3a, OsJIPR10*) or up-regulation (*OsFER2, OsMT3a*, *OsPR1b*) by treatment with high Fe (Fig. 2C).

Together, these findings indicated that treatment with Fe provokes transcriptional alterations in genes involved in iron homeostasis and defense responses in the absence of pathogen infection. Down regulation of defense-related genes by treatment with high Fe (e.g. *PR* genes) is in apparent contradiction with the phenotype of blast resistance that is observed in High-Fe plants. We then reasoned that blast resistance in rice plants that have been treated with high Fe might rely more on the stronger induction of defense responses upon pathogen infection rather than on constitutive expression of defense genes (see below).

### Transcriptional Reprogramming in Leaves of High-Fe Rice Plants During *M. oryzae* Infection

**Fig. 3:**
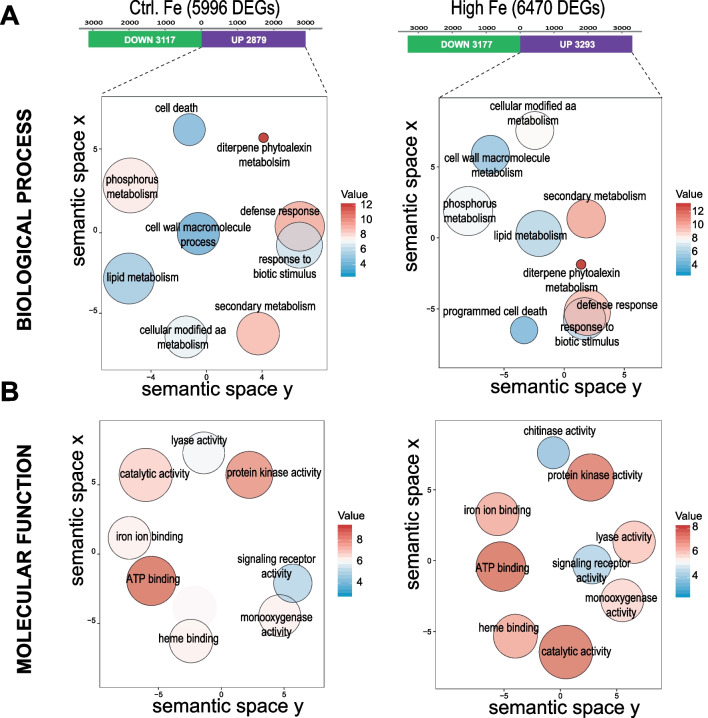
GO enrichment of genes up-regulated by *M. oryzae* infection in Control and High-Fe plants (48 hpi). The categories of Biological Processes (**A**) and Molecular Function (**B**). GO terms were visualized using REVIGO after reducing redundancy and clustering of similar GO terms in the *O. sativa* database. GO terms are represented by circles and are clustered according to semantic similarities (more general terms are represented by larger size circles, and adjoining circles are most closely related). Circle size is proportional to the frequency of the GO term, whereas color indicates the enrichment derived from the AgriGO analysis (red higher, blue lower). Full data sets of DEGs and lists of GO terms are presented in Additional file 3: Table S2 and Additional file 4: Table S3, respectively)

To explore the possibility that treatment of rice plants with high Fe has an effect on the induction of defense genes during pathogen infection, we compared the leaf transcriptomes of Control and High-Fe plants, inoculated with *M. oryzae* spores or mock-inoculated (at 48 h post-inoculation, hpi). The same criteria described above were used to identify pathogen-regulated genes in each Fe condition (log_2_ FC ≥ 0.5 or ≤  − 0.5 for up-regulated and down-regulated genes, respectively; *P* ≤ 0.05). Pair-wise comparisons of DEGs (*M. oryzae*-inoculated *vs* mock-inoculated) revealed 5996 genes regulated by *M. oryzae* infection in Control Fe plants (2879 up-regulated; 3117 down-regulated) (Fig. 3A, Additional file 3: Table S2a). In High-Fe plants, 6470 genes were regulated by *M. oryzae* infection (3293 up-regulated; 3177 down-regulated) (Fig. 3A, Additional file 3: Table S2b). Most of the genes regulated by pathogen infection, either up-regulated or down-regulated genes, were shared between Control and High-Fe plants (Additional file 1: Fig. S2).

Gene ontology (GO) enrichment analysis was used to identify GO terms enriched in the set of pathogen-regulated genes in High-Fe or Control plants. Redundant GO terms were removed using the REVIGO tool (Supek et al. 2011; http://revigo.irb.hr). In Biological Processes, the most abundant subcategories were “Diterpene Phytoalexin Metabolism” followed by “Secondary Metabolism” and “Response to Biotic Stimulus/Defense Responses”, which were enriched in both High-Fe and Control-Fe plants (Fig. 3A, Additional file 4: Table S3a, b). The GO category of “Phosphorus Metabolism” was also enriched in the set of genes that are up-regulated by pathogen infection (Fig. 3A). Indeed, an enrichment of GO terms for “ATP binding” and “Protein kinase activity” annotations were found in the Molecular Function category, both in High-Fe and Control plants (Fig. 3B). Genes involved in “Iron binding” and “Signaling receptor activity” were also over-represented in the set of up-regulated genes by pathogen infection in both High-Fe and Control plants, while genes with “Chitinase activity” were over-represented in High-Fe plants, but not in Control plants (Fig. 3B). In this respect, transgenic expression of chitinase genes has long been recognized to confer resistance to fungal diseases in different plant species, including rice blast disease (Ali et al. 2018). As for genes that are down-regulated by *M. oryzae* infection, in Biological Processes, they categorized in “Photosynthesis” and “Pigment biosynthetic processes” in both High-Fe and Control plants (Additional file 1: Fig. S3A). In Molecular Function, GO terms for “Iron-sulfur cluster binding” and “Zinc ion binding” were represented in the group of down-regulated genes in Control and High-Fe plants (Additional file 1: Fig. S3B).

Taken together, GO enrichment analysis of genes regulated by *M. oryzae* infection indicated similar GO terms in High-Fe and Control rice plants, indicating that similar biological processes and mechanisms appear to be activated by pathogen infection in both conditions. Then, quantitative rather than qualitative differences in defense gene expression would explain the phenotype of blast resistance that is observed in High-Fe plants. Indeed, hierarchical clustering of DEGs expression levels in Control and High-Fe plants (infected and mock) revealed that the transcriptional response to *M. oryzae* infection in High-Fe plants was not qualitatively different from that in Control plants, but quantitatively different (Additional file 1: Fig. S4).

**Fig. 4:**
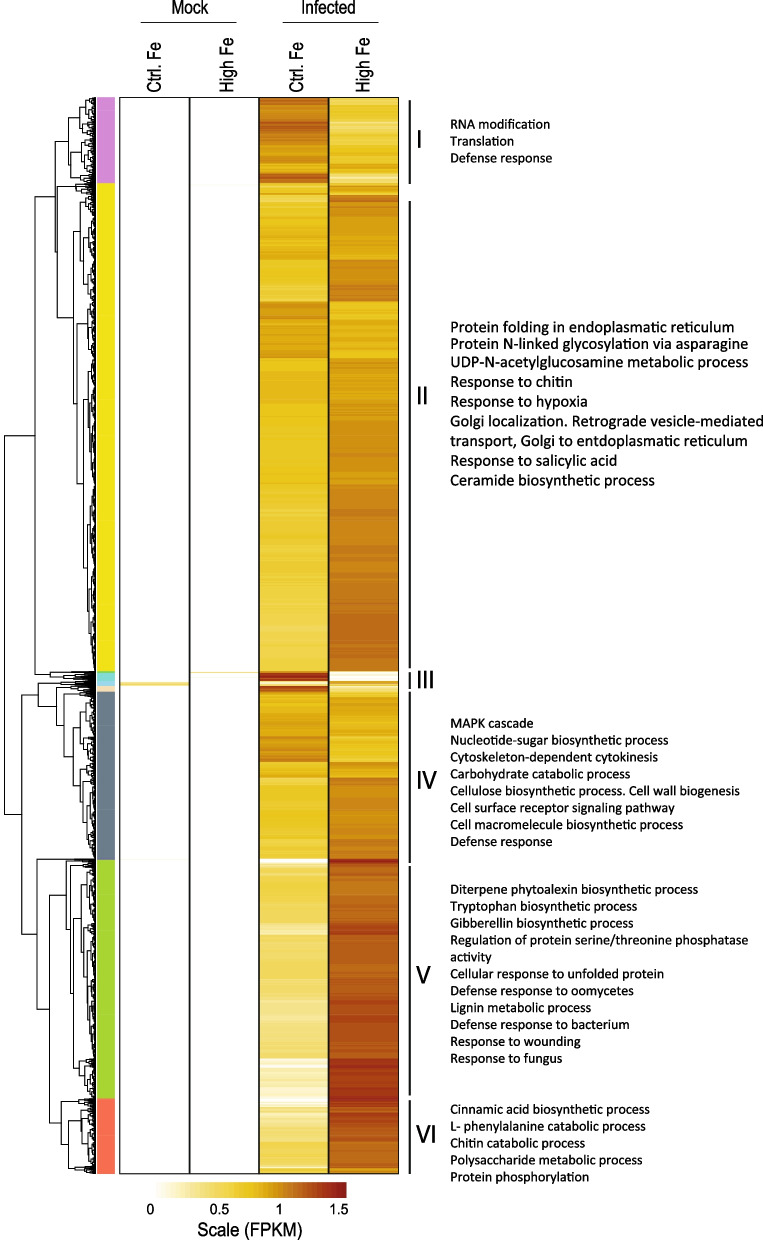
Hierarchical clustering of genes that are regulated by *M. oryzae* infection in Control and High-Fe plants. The expression level is depicted as row scaled FPKM (fragments per kilobase per million reads) and is represented from pale yellow (less expressed) to brown (more expressed). GO enrichment analyses of DEGs in each individual cluster revealed over-represented GO terms (shown on the right)

To further investigate differences in the response of Control and High-Fe plants to *M. oryzae* infection, we examined co-expression patterns. Genes up-regulated by *M. oryzae* infection classified into 6 hierarchical clusters (Fig. 4). GO terms in co-expressing genes identified terms related to plant defense in clusters I, II, IV, and V, whereas genes playing a role in protein phosphorylation grouped in cluster VI (Fig. 4). In particular, Cluster V contained the GO terms of “Diterpene phytoalexin biosynthetic process” and “Response to fungus/bacterium/oomycetes”. This analysis also revealed that High-Fe plants exhibited stronger induction in the expression of pathogen-responsive genes compared with Control plants (Fig. 4).

### Enhanced Defense Responses of Iron-Treated Rice Plants Against the Blast Fungus

**Fig. 5:**
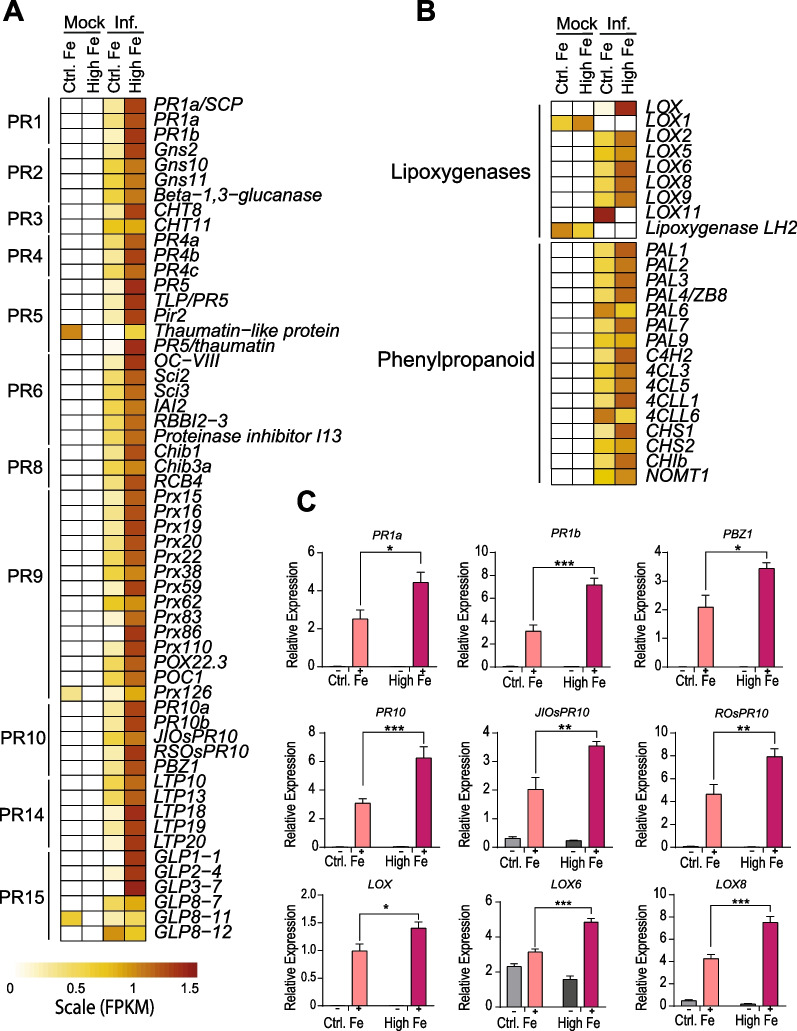
Expression of genes involved in defense responses in leaves of Control and High-Fe plants. Heat maps (A and B) show the expression pattern of DEGs identified by RNA-Seq analysis (Mock and infection conditions, 48 hpi) as row scaled FPKM (fragments per kilobase per million reads), from pale yellow (less expressed) to brown (more expressed). **A** Expression of *Pathogenesis-Related* (*PR*) genes. **B** Expression of lipoxygenase and phenylpropanoid genes. Gene names, locus IDs, FPKMs and fold change values are indicated in Additional file 5: Table S4. (**C**) Transcript levels were determined by RT-qPCR analysis (−, mock-inoculated; +, *M. oryzae*-inoculated). The expression values were normalized to the rice *Ubiquitin1* gene. Four independent biological replicates were examined. Data are mean ± SEM. Asterisks indicate statistical significance calculated by two-way ANOVA (*, *P* < 0.05; **, *P* < 0.01, *** *P* < 0.001). Gene-specific primers are listed in Additional file 9: Table S8

The induction of *PR* genes is regarded as a ubiquitous response to pathogen infection in plants. Presently, PR proteins are grouped into 17 families based on their protein sequence similarities and function (Ali et al. 2018). As expected, our RNASeq analysis revealed that *M. oryzae* infection activated the expression of an important number of *PR* genes (Fig. 5A, Additional file 5: Table S4). They included genes belonging to different *PR* families, such as *PR1, PR2* (β-1,3 glucanases)*, PR3, PR4* and *PR8* (chitinases)*, PR5* (thaumatin-like proteins, TLPs)*, PR6* (proteinase inhibitors)*, PR9* (peroxidases)*, PR10* (ribonuclease-like proteins), *PR14* (lipid transfer proteins, LTPs) and *PR15* (germin-like proteins) genes. Of them, *OsPR1* and *OsPBZ1* (*PR10* family) are routinely used as marker genes for the induction of the rice response to *M. oryzae* infection (Midoh and Iwata 1996; Agrawal et al. 2001)**.** Although *PR* gene expression was induced by *M. oryzae* infection in both High-Fe and Control plants, their expression was induced to a greater extent in High-Fe plants compared with Control plants (Fig. 5A, Additional file 5: Table S4). Here it is worth mentioning that transgenic expression of distinct *PR* genes, such as chitinase or *PR10* genes, has proven to enhance resistance to *M. oryzae* infection (Wu et al. 2016; Ali et al. 2018).

RNA-Seq analysis also revealed regulation of an important number of peroxidase (*Prx*) genes during *M. oryzae* infection (*PR9* family), these genes exhibiting stronger induction in High-Fe plants compared with Control plants (Fig. 5A, Additional file 5: Table S4). It is tempting to hypothesize that *M. oryzae*-regulated peroxidases might contribute to control the spatial and temporal accumulation of H_2_O_2_ in rice plants.

Among the genes regulated by both Fe treatment and *M. oryzae* infection there were several lipoxygenases (Fig. 5B). Lipoxygenases (LOXs) are crucial for lipid peroxidation processes during plant defense responses to pathogen infection. For instance, LOX are involved in the conversion of polyunsaturated fatty acids into oxylipins and jasmonates (Viswanath et al. 2020). Lipoxygenases have been also implicated in the initiation and execution of ferroptotic cell death in plants (Conrad et al. 2018).

Defensive functions of phenylpropanoid compounds have long been recognized, these metabolites conferring broad-spectrum disease resistance in different plant species (Yadav et al. 2020). To note, genes involved in the general phenylpropanoid pathway were found to be regulated by both iron treatment and *M. oryzae* infection, most of these genes showing a stronger response in High-Fe plants than in Control plants (Fig. 5B, Additional file 5: Table S4). A schematic representation of these genes in the rice phenylpropanoid pathway is presented in Additional file 1: Fig. S5. They included genes that participate in the general phenylpropanoid pathway (*phenylalanine ammonia lyase, PAL*; *cinnamic acid 4-hydroxylase, C4H*; *4-coumarate:CoA ligase, 4CL*), as well as genes in branches leading to flavonols and anthocyanins (*chalcone synthase, CHS*; *chalcone isomerase, CHI*). The phenylpropanoid pathway also leads to the production of naringenin, the precursor of flavonols. Naringenin is also the precursor molecule for sakuranetin, the only flavonoid phytoalexin so far identified in rice (Hasegawa et al. 2014). Sakuranetin has been shown to accumulate in rice leaves during *M. oryzae* infection and to exhibit antifungal activity against *M. oryzae* (Hasegawa et al. 2014). We noticed that *OsNOMT1* (Naringenin 7-*O*-methyltransferase), responsible of methylation of naringenin to sakuranetin (Shimizu et al. 2012) reached a higher expression in High-Fe compared with Control plants (Fig. 5B). RT-qPCR confirmed higher expression levels of defense-related genes in response to pathogen infection in High-Fe plants compared with infected Control plants (*OsPR1a*, *OsPR1b*, *OsPBZ1, PR10*, *JIOsPR10, ROsPR10, OsLOX*, *OsLOX6*, *OsLOX8*) (Fig. 5C).

Altogether, these results indicated that treatment with high Fe is associated with a stronger induction of defense gene expression during *M. oryzae* infection, which correlates well with resistance to *M. oryzae* in High-Fe plants.

### Treatment with High Fe Promotes the Accumulation of Phytoalexins in Rice Leaves

Phytoalexins are low-molecular-weight antimicrobial compounds that are produced by plants in response to pathogen infection (Ahuja et al. 2012). Major phytoalexins in rice accumulating during *M. oryzae* infection are diterpene phytoalexins and the flavonoid phytoalexin sakuranetin (Okada et al. 2007; Hasegawa et al. 2010, 2014).

**Fig. 6:**
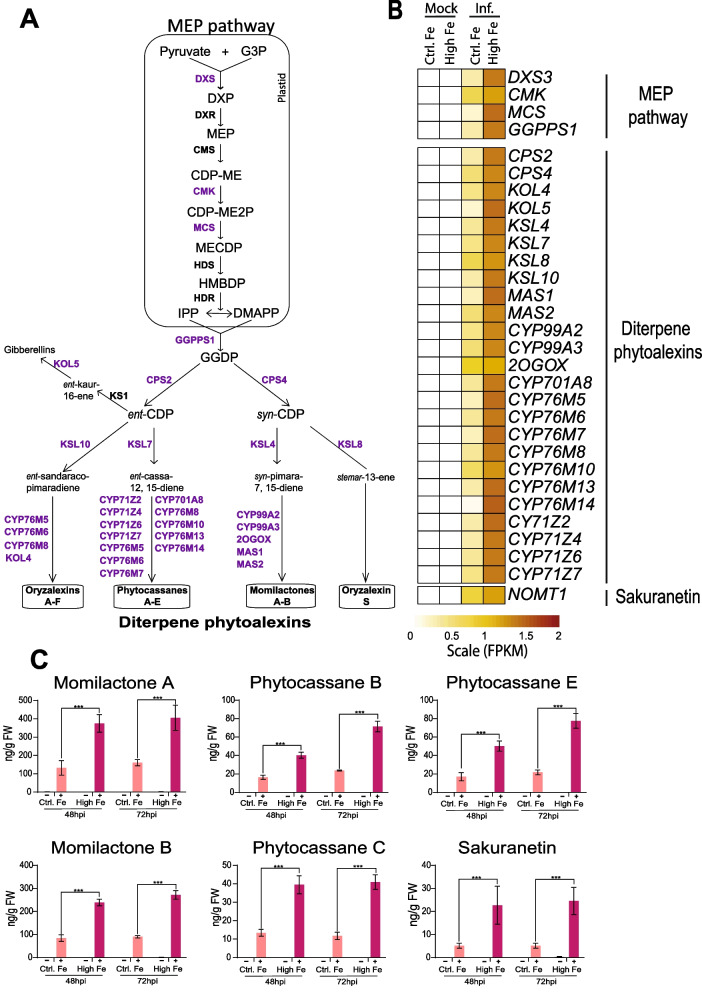
Accumulation of phytoalexins in leaves of rice plants that have been grown under high Fe supply. **A** Methylerythritol phosphate (MEP) and diterpenoid phytoalexin biosynthesis pathways in rice. Genes whose expression is up-regulated by *M. oryzae* infection in control and high-Fe plants are indicated in purple color. The full name and details on the expression of these genes can be found in Additional file 6: Table S5. **B** Heat map showing expression levels (row scaled FPKM, fragments per kilobase per million reads) in leaves of Control and High-Fe plants, mock-inoculated and *M. oryzae*-inoculated plants (48hpi). Gene expression is represented from pale yellow (less expressed) to brown (more expressed). **C** Accumulation of phytoalexins, diterpenoid phytoalexins and sakuranetin, in leaves of Control and High-Fe plants at 48 hpi and 72 hpi with *M. oryzae* spores: momilactones (A and B), phytocassanes (B, C, and E), and the flavonoid phytoalexin sakuranetin (−, mock-inoculated; +, *M. oryzae*-inoculated). Data are mean ± SEM of three biological replicates each with five plants. Asterisks indicate statistically significant differences calculated by two-way ANOVA (*** *P* ≤ 0.001). G3P, glyceraldehyde-3-phosphate; DXP, 1-deoxy-D-xylulose 5-phosphate; MEP, 2- C-methyl-D-erythritol 4-phosphate; CDP-ME, 4-(cytidine 5′-diphospho)-2-C-methyl-D-erythritol; CDP-ME2P, 2-phospho-4-(cytidine 5′- diphospho)-2-C-methyl-D-erythritol; MECDP, 2-C-methyl-D-erythritol 2,4-cyclodiphosphate; HMBDP, 1-hydroxy-2-methyl-2-(E)-butenyl 4-diphosphate; IPP, isopentenyl diphosphate; DMAPP, dimethylallyl diphosphate; GGDP, geranylgeranyl diphosphate; and CDP, copalyl diphosphate

Diterpenoid phytoalexins are synthesized from geranylgeranyl diphosphate (GGDP), the end product of the methylerythritol phosphate (MEP) pathway (Fig. 6A). Rice produces a variety of diterpene phytoalexins, namely momilactones (A, B), phytocassanes (A to E), and oryzalexins (A to F, S). Our RNA-Seq analysis revealed that the expression of genes implicated in the MEP and diterpene phytoalexin biosynthesis pathways was induced to a greater extend in High-Fe plants than in Control plants (Fig. 6B, Additional file 6: Table S5). They included genes in the biosynthetic pathway leading to the production of oryzalexins (A–F), phytocassanes (A–E) and momilactones (A and B) whose expression was strongly induced by pathogen infection in High-Fe plants compared with Control plants (Fig. 6B, Additional file 6: Table S5). Results obtained by RNA-Seq analysis were confirmed by RT-qPCR analysis for selected genes (Additional file 1: Fig. S6). As previously mentioned, RNA-Seq analysis also revealed stronger induction of *OsNOMT1* involved in sakuranetin (phenolic phytoalexin) biosynthesis in the response of High-Fe plants to *M. oryzae* infection compared with Control plants (Fig. 6, Additional file 6: Table S5).

Next, we investigated whether superinduction of phytoalexin biosynthesis genes in High-Fe plants during infection has an effect on phytoalexin content. As shown in Fig. 6C, M*. oryzae* infection is accompanied by the accumulation of diterpene phytoalexins (Momilactone A and B, Phytocassane B, C and E) in leaves of Control and High-Fe plants. Most importantly, their accumulation was found to be significantly higher in High-Fe plants than in Control plants (Fig. 6C). Equally, the phenolic phytoalexin Sakuranetin accumulated at a higher level during infection of High-Fe plants compared with Control plants (Fig. 6C). These results are in agreement with those obtained on the expression of phytoalexin biosynthetic genes indicating that treatment with high-Fe is associated to a higher accumulation of phytoalexins in rice plants, both diterpene and phenolic phytoalexins.

Phytoalexin content was also measured in rice plants grown under Fe limiting conditions. For this, the rice plants were grown for 16 days under control Fe conditions followed by 5 days under low Fe (Low-Fe) or Control Fe condition (Ctrl-Fe), and then inoculated with *M. oryzae* spores (Additional file 1: Fig. S7A, left panel). As expected, treatment with high Fe results in a decrease in total Fe content in rice tissues (Additional file 1: Fig. S7A, right panels) Under these experimental conditions, there were no statistically significant differences in phytoalexin content (momilactone A, momilactone B, phytocassane C, phytocassane B, phytocassane E sakuranetin) in *M. oryzae*-infected Low Fe plants compared with *M. oryzae*-infected Control plants (Additional file 1: Fig. S7B).

Knowing that rice phytoalexins have been described to exhibit antifungal activity against *M. oryzae* (Umemura et al. 2003; Hasegawa et al. 2010, 2014), it is tempting to hypothesize that blast resistance in High-Fe plants might result, as least in part, from the superactivation of phytoalexin biosynthesis genes, and subsequent accumulation of phytoalexins in these plants.

### *M. oryzae* Infection Modulates Iron Accumulation in Rice Leaves

**Fig. 7:**
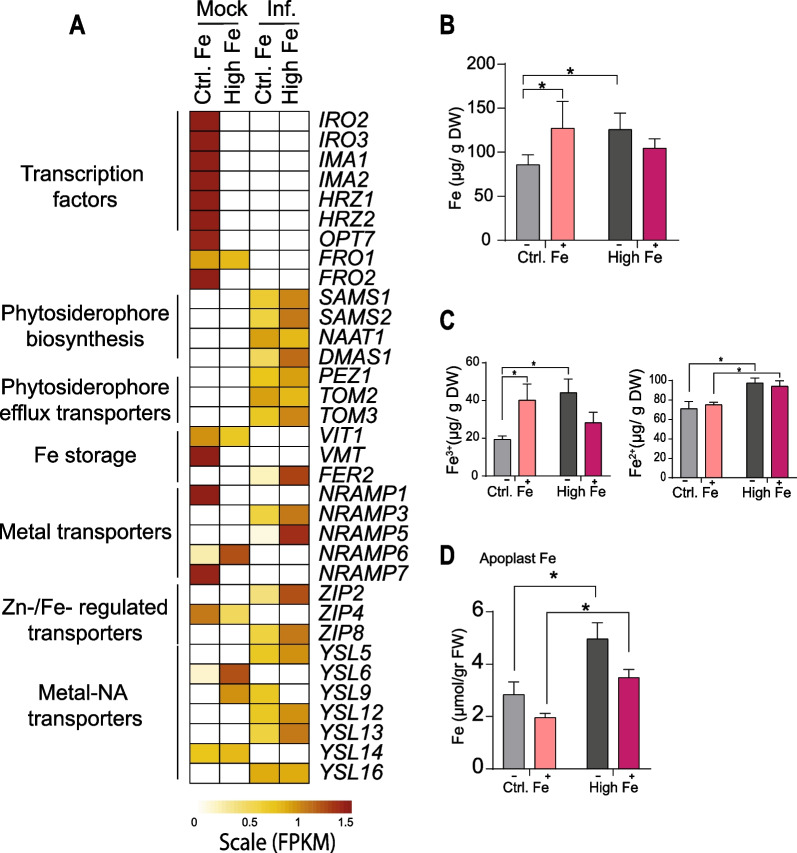
Effect of pathogen infection on Fe homeostasis-related genes and pathogen-induced alterations on Fe content in leaves. **A** Heat map showing the expression level of iron-related genes (row scaled FPKMs) in leaves of Control and High-Fe plants (mock-inoculated and *M. oryzae*-inoculated) at 48 hpi. Gene expression is represented from pale yellow (less expressed) to brown (more expressed). The full name and details on the expression of these genes are indicated in Additional file 7: Table S6. **B** Total Fe content in the youngest developed leaf (third leaf) (−, mock-inoculated; +, *M. oryzae*-inoculated). Five independent biological replicates (three technical replicate each) were analyzed. **C** Fe^2+^ and Fe^3+^ content was determined by the colorimetric Ferrozine method in the youngest developed leaf of Control and High-Fe plants. **D** Fe content in the apoplast of leaves (youngest developed leaves) determined by ICP-MS. Five independent biological replicates (three technical replicate each) were analyzed. Data are mean ± SEM. Asterisks indicate statistically significant differences calculated by two-way ANOVA *, *P* ≤ 0.05). DW, dry weight; FW, fresh weight

Comparative transcriptome analysis of Control and High-Fe plants revealed that, in addition to defense-related genes, *M. oryzae* infection has an impact on the expression of genes that function in iron homeostasis in leaves. Among them, there were genes that are down-regulated by Fe treatment (e.g. in the absence of pathogen infection) that are also down-regulated by pathogen infection in Control plants. This is the case of the transcription factors *OsIRO2*, *OsIRO3*, *OsIMA1*, *OsIMA2*, *OsHRZ1, OsHRZ2*, the *OsOPT7 Oligopeptide transporter*, and *Ferric Reductase Oxidase 2* (*OsFRO2*) (Fig. 7A, Additional file 7: Table S6).

As chelators of Fe, rice plants produce phytosiderophores of the mugineic acid (MA) family, DMA (2-deoxymugineic acid) being the sole MA produced in rice. DMA is synthesized from S-adenosyl-methionine (SAM). To note, *OsSAMS1*, *OsSAMS2* (encoding SAM synthases 1 and 2) responsible for the conversion of L-methionine to SAM were found to be strongly up-regulated by pathogen infection in High-Fe plants (Fig. 7A). A priori, the up-regulation of these genes would ensure a sufficient supply of SAM for DMA conversion. Other genes in the biosynthesis of DMA that are regulated by *M. oryzae* infection were *OsNAAT1* (nicotianamine aminotransferase 1) and *OsDMSA1* (deoxymugineic acid synthase 1). Phytosiderophore efflux transporter genes (*OsTOM2, OsTOM3*) were also up-regulated by infection, these genes being implicated in the transport of DMA out of the cell (Fig. 7A).

Regarding vacuolar iron transporters, we found that the Vacuolar Iron Transporter 1 (*OsVIT1*) involved in Fe sequestration into vacuoles, was repressed during infection in both Control and High-Fe plants. As for the Vacuolar Mugineic acid Transporter (*OsVMT*) responsible for sequestration of DMA into the vacuoles, its expression was down-regulated by infection only in Control plants (Fig. 7A). To note, *M. oryzae* infection strongly induced *FERRITIN2* (*FER2*) in High-Fe plants (Fig. 7A). As previously mentioned, Ferritin is considered to be the major Fe-storage protein in plants.

Other Fe-related genes that are also regulated by pathogen infection were those involved in Fe transport, such as members of the *NRAMP* (*Natural Resistance-Associated Macrophage Protein*) family (*OsNRAMP1*, *OsNRAMP3*, *OsNRAMP5*, *OsNRAMP6*, *OsNRAMP7*), and the ZIP (Zn-regulated transporter, IRT-like protein) family (*OsZIP2*, *OsZIP4*, *OsZIP8*) (Fig. 7A). Additionally, up to 7 genes encoding Yellow Stripe-Like (YSL) transporters functioning in the import of Fe^3+^-DMA complexes into the plant cell were found to be regulated during *M. oryzae* infection in rice leaves. They were: *OsYSL5, OsYSL12*, *OsYSL13, OsYSL16* (up-regulated), *OsYSL6*, *OsYSL14* (down-regulated), and *OsYSL9* (up-regulated in Control plants but down-regulated in High-Fe plants) (Fig. 7A). Changes in the expression of selected iron homeostasis-related genes in response to *M. oryzae* infection in High-Fe and Control plants were confirmed by RT-qPCR analysis (Additional file 1: Fig. S8).

Altogether, this study revealed that *M. oryzae* infection triggers alterations in the expression of genes with different functions in maintenance of iron homeostasis, including transcriptional regulators of Fe-responsive genes, Fe transporters, and Fe chelators. Presumably, these genes would be of importance in maintaining proper spatio-temporal accumulation of Fe in the host tissue during pathogen infection.

Having established that pathogen infection regulates the expression of genes involved in Fe homeostasis, we determined whether *M. oryzae* infection provokes alterations in Fe content in rice leaves. Total Fe content was determined by inductively coupled plasma-mass spectrometry (ICP-MS) in leaves of Control and High-Fe plants, that have been inoculated with *M. oryzae* spores or mock-inoculated. As expected, treatment with high Fe results in an increase in total Fe content (Fig. 7B, grey bars). Upon pathogen challenge, there was a significant increase in Fe content in leaves of Control plants, but its level was not significantly altered in leaves of High-Fe plants (a tendency to a lower content in response to *M. oryzae* infection could be, however, observed in High-Fe plants) (Fig. 7B).

Next, it was of interest to investigate whether *M. oryzae* infection has an effect on the accumulation of each Fe ion in rice leaves, Fe^3+^ and Fe^2+^. Accordingly, the ferrozine assay was used to assess Fe^3+^ and Fe^2+^ accumulation in leaves of Control and High-Fe plants, mock-inoculated and *M. oryzae*-inoculated. Treatment with high Fe increased both Fe^3+^ and Fe^2+^ content in mock-inoculated plants (Fig. 7C, grey bars). Upon pathogen infection, Fe^3+^ content increased in Control plants, while its level decreased in High-Fe plants (infected *vs* non-infected plants) (Fig. 7C, left panel, pink bars), a pattern that resembled that of total Fe content (see Fig. 7B). From these results, it appears that Fe^3+^ content in *M. oryzae*-infected High-Fe plants reached a level similar to that in non-infected Control plants. As for Fe^2+^, its level was not significantly altered by pathogen infection either in Control or High-Fe plants (Fig. 7C, right panel).

To gain more information about effect of pathogen infection on Fe distribution in rice leaves, we measured apoplastic Fe levels. Malate dehydrogenase (MDH) activity, an indicator of cytosolic contamination, was not detected in apoplast samples, thus, excluding cytosolic contamination in apoplastic samples (Additional file 1: Fig. S9). This study revealed that treatment with high-Fe was accompanied by a significant increase in apoplastic Fe concentrations (Fig. 7D, grey bars). Upon *M. oryzae* infection, apoplastic Fe decreased in both Control and High-Fe plants (Fig. 7D, pink bars). In this way, *M. oryzae* infection decreased Fe accumulation in the leaf apoplast of High-Fe plants to a level similar to that in non-infected Control plants.

Collectively, transcriptome analysis in combination with Fe determinations revealed that *M. oryzae* infection provokes alterations in the expression of genes involved in Fe homeostasis leading to alterations in Fe content, total Fe and apoplastic Fe, in rice leaves. Different responses to pathogen infection are, however, observed depending on the Fe supply condition. In High-Fe plants, there is a reduction on total Fe, Fe^3+^ and apoplastic Fe during pathogen infection, whereas pathogen infection increases total and Fe^3+^ content, but not apoplastic Fe, in control plants. Clearly, a tight control of Fe homeostatic mechanisms occurs in rice plants at the intracellular and extracellular levels during infection by *M. oryzae*, which is also dependent on the Fe status of the rice plant.

### ROS and Fe Accumulation in High-Fe Plants During Pathogen Infection

The production of ROS is a generalized plant defense response against pathogen attack (Torres 2010). Among ROS, H_2_O_2_ is considered to be an important signaling molecule in regulating plant immunity. It is also known that pathogen infection triggers ROS accumulation and localized cell death around the site of infection, thus, limiting the spread of the pathogen. Additionally, Fe-mediated ROS production (Fenton reaction) is a central executioner of ferroptosis, an iron-dependent form of cell death which is also associated with increased lipid peroxidation (Distéfano et al. 2017; Conrad et al. 2018). The observation that *M. oryzae* infection was accompanied by alterations in Fe accumulation in rice leaves prompted us to investigate Fe and ROS accumulation in leaves of High-Fe plants during *M. oryzae* infection at the histochemical level.

**Fig. 8:**
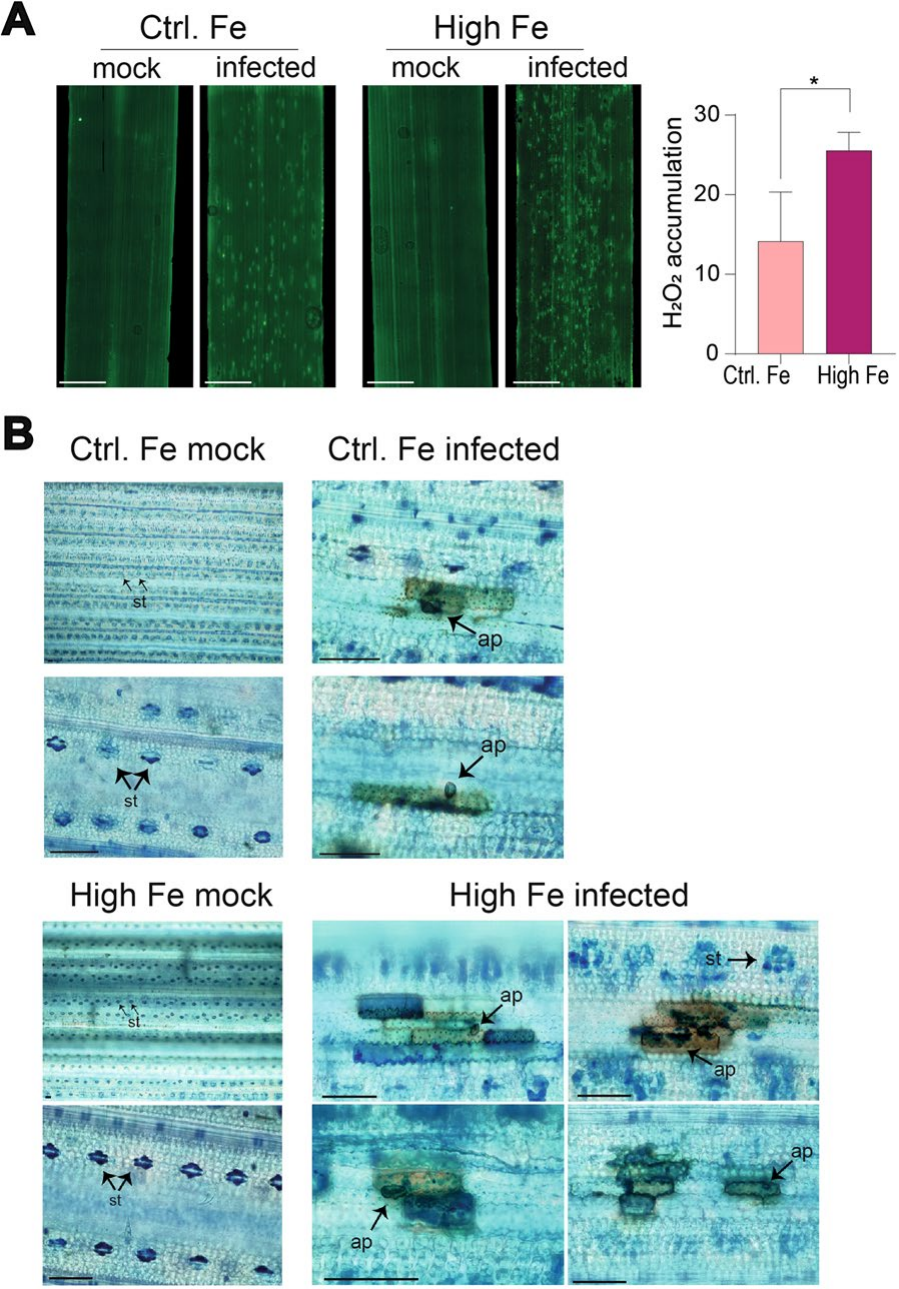
Accumulation of ROS and Fe in leaves of Control and High-Fe rice plants. **A** ROS (H_2_O_2_) was detected in leaves using the fluorescent probe H_2_DCFDA. Leaves (youngest developed leaves) from mock-inoculated (mock) and *M. oryzae*-inoculated plants (48 hpi) were examined. Bars correspond to 2 mm. Right panel, quantification of ROS fluorescence using Image J software (three independent experiments, 100 fields, each). Asterisks indicate statistically significant differences calculated by two-way ANOVA (*P ≤ 0.05). **B** Fe detection in leaves of Control-Fe (upper panels) and High-Fe (lower panels) plants that have been mock-inoculated or infected with *M. oryzae* (48 hpi). The youngest developed leaf (third leaf) was subjected to DAB staining (brown) followed by Perls staining (blue). Bars correspond to 50 μm. St, stomata, Ap: appressorium

Histochemical detection of ROS was carried out using the fluorescent probe H_2_DCFDA (2′, 7′ dichlorofluorescein diacetate) which mainly detects H_2_O_2_ accumulation (Fichman et al. 2019). In the absence of *M. oryzae* infection, ROS was not detected in leaves of either Control or High-Fe plants (Fig. 8A, mock). Upon pathogen challenge, however, discrete regions accumulating ROS could be observed in both Control and High-Fe plants, but the H_2_DCFDA-fluorescent signals were more intense and abundant in High-Fe plants relative to Control plants (Fig. 8A, infected). Quantification of H_2_DCFDA fluorescence using ImageJ software confirmed higher pathogen-induced accumulation of ROS in High-Fe plants compared with Control plants (Fig. 8A, right panel). Regions accumulating ROS, most probably, correspond to the *M. oryzae* infection sites.

To further investigate Fe accumulation during infection of High-Fe plants we used an histochemical procedure based on 3, 3′-diaminobenzidine (DAB) staining followed by Perls staining. In mock-inoculated leaves from Control and High Fe plants, Fe^3+^ ions were detected in stomata (Fig. 8B, blue color). Upon *M. oryzae* infection of Control-Fe plants, ROS was found to accumulate at the invaded leaf cells (Fig. 8B, brown color; Ctrl. Fe infected). Interestingly, in *M. oryzae*-infected High Fe plants, ROS (brown) and Fe^3+^ ions (blue) accumulated at the infection sites with the surrounding cells also accumulating Fe^3+^ ions (Fig. 8B, High Fe infected).

Thus, transcriptome analysis in combination with histochemical detection of iron indicated that pathogen infection causes a redistribution of Fe in the rice leaf. Fe preferentially accumulates around the infection sites, most probably, as a consequence of alterations in the expression of genes involved in iron homeostasis-related genes during pathogen infection. The higher content of Fe in leaves of High-Fe plants would contribute to a higher accumulation of Fe (hence, ROS) at the infection sites in High-Fe plants. Alterations in iron distribution have been previously described in Arabidopsis tissues infected by *Dickeya dadantii,* and in maize plants infected by *Curvularia lunata* (Aznar et al. 2014; Fu et al., 2022). However, additional investigation needs to be carried out to determine the precise spatio-temporal accumulation of Fe during *M. oryzae* infection at both tissue and subcellular levels.

Finally, trypan blue staining revealed a pattern of cell death in leaves of *M. oryzae*-infected High-Fe plants that was absent in leaves of *M. oryzae*-infected Control plants (Additional file 1: Fig. S10). A closer examination of these regions revealed dead cells clustered in the vicinity of fungal penetration sites (appressoria) in the *M. oryzae*-infected High-Fe plants (Additional file 1: Fig. S10). These results confirmed that cell death occurs during infection of Fe-treated rice plants.

### Treatment with the Ferroptosis Inhibitor Ferrostatin-1 Suppresses Fe Accumulation During *M. oryzae* Infection

**Fig. 9:**
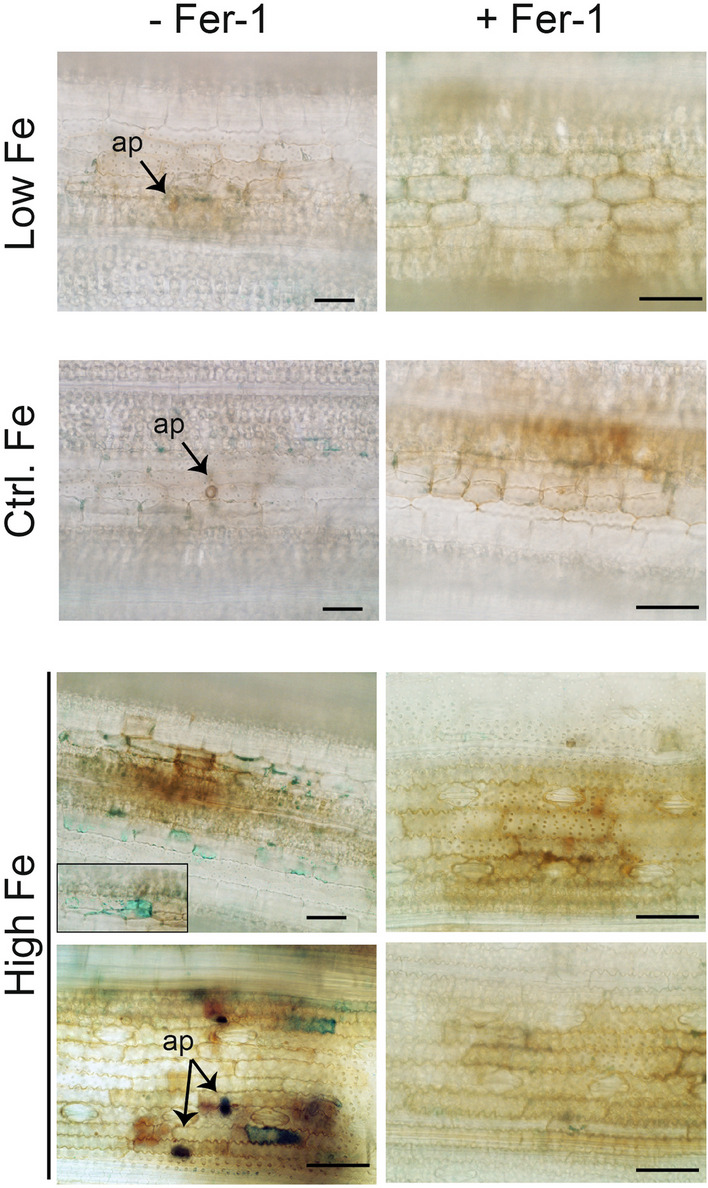
Fe detection in leaves of Low-Fe, Control-Fe, and High-Fe rice plants that have been inoculated with *M. oryzae* spores (48 hpi). Leaves were treated with the ferroptosis inhibitor Ferrostatin (Fer-1) (+ Fer-1) or not (− Fer-1). Rice leaves (youngest developed leaves) were subjected to Perls staining for detection of Fe (blue color). The images shown are representative of two independent experiments. Bars correspond to 100 µm

The ferroptosis inhibitor Ferrostatin-1 (Fer-1) was used to examine Fe accumulation during infection of rice plants that have been grown under high, control or low Fe supply. Perls staining of *M. oryzae*-infected leaves indicated that treatment with Fer-1 suppresses Fe accumulation at the infection sites, hence ferroptosis (Fig. 9). Brown spots observed in non-treated High-Fe plants (-Fer-1) points to a HR reaction in these plants. Treatment with Fer-1 of *M. oryzae*-infected leaf sheaths gave similar results. Here, Fe accumulation was also suppressed upon infection with *M. oryzae* (Additional file 1: Fig. S11). These findings confirm ferroptosis mediating blast resistance in High-Fe rice plants. In other studies, the use of ferroptosis inducers was found to prevent invasive growth of *M. oryzae* in leaf sheaths of an otherwise susceptible *indica*-type rice cultivar (*O. sativa* cv CO 39) (Shen et al. 2020).

**Fig. 10:**
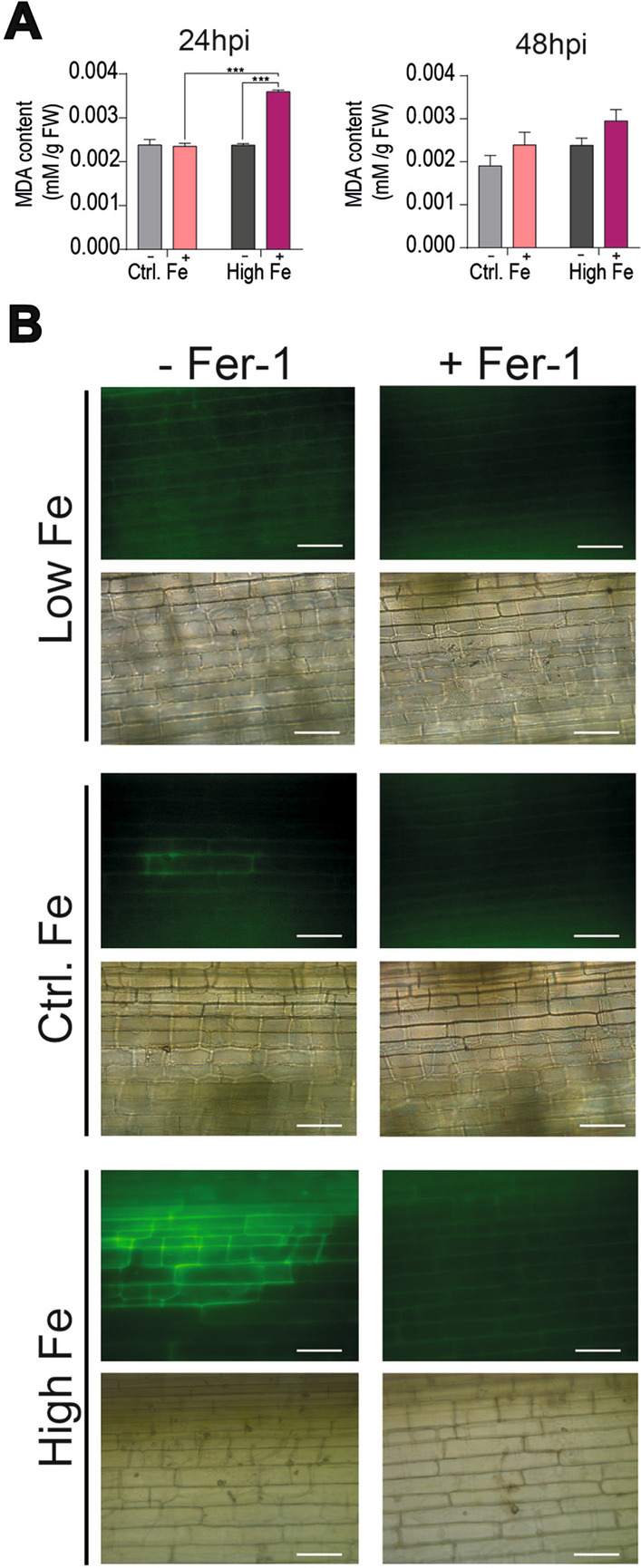
Lipid peroxidation in leaves of Low-Fe, Control-Fe, and High-Fe rice plants upon infection with *M. oryzae*. **A** MDA content in leaves (youngest developed leaves) of mock-inoculated and *M. oryzae*-inoculated plants (−, mock-inoculated; +, inoculated with *M. oryzae* spores). **B** Confocal microscopy was performed on *M. oryzae*-infected leaves (48 hpi) treated, or not, with the ferroptosis inhibitor Fer-1, and stained with C11-BODIPY^581/591^ to monitor oxidized lipid peroxides. Lower panels show the corresponding bright field images. Representative results from two independent experiments are shown. Ap, Appressorium. Bars correspond to 50 µm

As noted above, lipid peroxidation is also a hallmark of ferroptosis which is mainly caused by iron-mediated ROS accumulation (Yang et al. 2016; Conrad et al. 2018). In this work, we measured lipid peroxidation as malondialdehyde (MDA) content, MDA being a typical breakdown product of peroxidized fatty acids in plant membranes. Under no infection conditions, the MDA levels were similar in Control and High-Fe plants (Fig. 10A, grey bars). Under infection conditions, however, MDA accumulated at a higher level in High-Fe plants than in Control plants in response to pathogen infection, a response that was more evident at 24 hpi (Fig. 10A, pink bars).

To further investigate lipid peroxidation in High-Fe plants, we used C11-BODIPY^581/591^ (from now on BODIPY), a fluorescent lipid peroxidation reporter molecule that allows monitoring lipid peroxidation in cell membranes associated to pro-oxidant activity during ferroptosis (upon oxidation, BODIPY shows green fluorescence). As shown in Fig. 10B (left panels), leaves of High Fe plants showed the characteristic green fluorescence of oxidized BODIPY at the infected regions, which was not observed in *M. oryzae*-infected leaves of either Control plants or Low-Fe plants. Treatment with Fer-1 showed a near-complete loss of accumulation of oxidized BODIPY (Fig. 10B, right panels). These findings further support lipid peroxidation and ferroptosis during *M. oryzae* infection in leaves of High-Fe plants.

Collectively, results here presented indicated that rice plants that have been exposed to treatment with high Fe develop a ferroptotic cell death during infection with a virulent isolate of *M. oryzae*, a response that also occurs during infection of rice leaf sheaths with an incompatible *M. oryzae* isolate (Dangol et al. 2019).

## Discussion

In this study, we provide evidence that short-term exposure to iron enhances resistance to infection by the blast fungus *M. oryzae* in rice plants. Pathogen resistance in Fe-treated rice plants is associated with stronger induction of defense responses during pathogen infection, including higher production of ROS and superinduction of *PR* genes. Treatment with high Fe also provokes superactivation of genes for the biosynthesis of phytoalexins during pathogen infection, both diterpene phytoalexins and sakuranetin. This, in turn, results in higher accumulation of phytoalexins in *M. oryzae*-infected leaves of High-Fe plants. Knowing that rice phytoalexins exhibit antifungal activity against *M. oryzae* (Okada et al. 2007; Hasegawa et al. 2014), a higher accumulation of phytoalexins would explain, at least in part, the phenotype of blast resistance in High-Fe plants. In previous studies, it was described that growing rice plants in low Fe increases blast susceptibility (Peris-Peris et al. 2017). The observed pathogen-induced transcriptional alterations in High-Fe plants and Control plants were not qualitatively different each other, but the magnitude of change in gene expression was greater in High-Fe plants compared with that in Control plants. Overall, these findings support priming for enhanced defense in Fe-treated rice plants. Defense priming has been shown to occur in plants upon treatment with natural or synthetic compounds (Martinez-Medina et al. 2016). In a more general sense, these findings indicated that Fe can regulate the expression of a range of genes through which rice immunity is controlled.

Histochemical staining revealed that, under non-infection conditions, Fe preferentially accumulates at stomata whereas during pathogen infection, Fe accumulates in cells that are in the proximity to the infection sites. Most probably, a redistribution of Fe occurs in the leaf tissue during pathogen infection. Knowing the capacity of Fe to produce ROS and the general requirement of ROS production for the induction of defense responses, the accumulation of Fe, and subsequent ROS production at the infection sites might well trigger the activation of immune responses in High-Fe plants (Distéfano et al. 2021). Trypan blue staining confirmed cell death in cells located in the vicinity of the sites of fungal penetration in the host tissue. The phenotype of cell death induced by treatment with high Fe closely resembles the hypersensitive response induced by infection with an avirulent pathogen which would explain the phenotype of blast resistance that is observed in High-Fe plants. Also, ROS accumulation at the sites of infection can be potentially toxic to the pathogen while avoiding Fe toxicity caused by a generalized accumulation of Fe.

Several lines of evidence support that ferroptosis contributes to blast resistance in a susceptible rice cultivar that has been subjected to short-term treatment with high Fe. As previously mentioned, the accumulation of Fe-dependent ROS is a hallmark of ferroptosis, a phenomenon that is observed in *M. oryzae*-infected High-Fe plants. In addition to Fe-dependent ROS production, lipid peroxidation is also common feature for ferroptosis. In this respect, several lipoxygenases were found to be strongly induced in High-Fe plants, these plants also accumulating higher levels of MDA than Control plants during *M. oryzae* infection. Treatment with the canonical ferroptosis inhibitor Fer-1 prevented Fe accumulation and lipid peroxidation in High-Fe plants which further supports ferroptosis during infection of High-Fe rice plants.

In plants, ferroptosis was first reported in *Arabidopsis thaliana* under heat stress conditions (Distéfano et al. 2017). Later on, Dangol et al. (2019) reported ferroptosis occurring during infection of rice plants with an avirulent *M. oryzae* strain (incompatible interaction), but not upon infection with a virulent strain of *M. oryzae.* In this way, ferroptosis appears to occur not only in incompatible rice/*M. oryzae* interactions (Dangol et al. 2019) but also in susceptible rice plants upon Fe treatment (present work), or by treatment of susceptible plants with ferroptosis inducers (Shen et al. 2020).

In addition to defense-related genes, our results indicated that genes that are known to be involved in iron homeostasis were also regulated by *M. oryzae* infection. They included genes with functions related to the chelation/transport of Fe, import/export of Fe between subcellular compartments (plasma membrane, tonoplast), or Fe sequestration (e.g. Ferritin). As ferritin is known to bind Fe to avoid cellular Fe toxicity (Aung and Masuda 2020), it is tempting to hypothesize that ferritin might sequester intracellular Fe caused by pathogen infection in High-Fe plants, if in excess. In other studies, transgenic tobacco plants constitutively expressing alfalfa ferritin were more resistant to infection by fungal pathogens (*Alternaria alternata, Botrytis cinerea*) (Deák et al. 1999). Other genes playing a role in iron homeostasis that are also regulated by *M. oryzae* infection were *ZIP*, *YSL,* and *NRAMP* genes. Together, these findings support that plants have evolved mechanisms to sustain Fe homeostasis and immune responses in concerted action. The iron-regulated and pathogen-regulated genes identified in this study are good candidates to be components of regulatory connections between Fe signaling and immune signaling in resistance to infection by the blast fungus in rice.

We show that whereas *M. oryzae* infection causes an increase in Fe content (attributable to an increase in Fe^3+^) in Control Fe, non-treated rice plants, the level of apoplastic Fe is reduced in Control Fe plants during *M. oryzae* infection. On the other hand, in High-Fe rice plants, total Fe (and Fe^3+^) as well as apoplastic Fe content decreased in response to *M. oryzae* infection, thus, reaching levels similar to those in non-infected Control Fe plants. While Fe^3+^ levels are regulated by *M. oryzae* infection, Fe^2+^ accumulation appears not to be affected by pathogen infection, either in Control- or High-Fe plants. A redistribution of intracellular and apoplastic Fe, as well as changes in the spatial and temporal accumulation of Fe, might well be the consequence of pathogen-induced alterations in the expression of genes involved in iron homeostasis. Thus, a regulatory network of iron chelation, transport and trafficking operating in *M. oryzae*-infected rice leaves would facilitate recruitment of Fe towards the infection sites (as revealed by histochemical analysis in leaves of *M. oryzae*-infected plants). Whether mobilization of Fe from non-infected leaf regions towards the infection sites occurs during infection is an interesting issue that deserves further investigation.

Several studies gathered evidence of connections between the Fe status of plants and disease resistance (Kieu et al. 2012; Verbon et al. 2017; Herlihy et al. 2020). For instance, Fe deficiency was found to confer resistance to *Dickeya dadantii* and *Botrytis cinerea* in Arabidopsis (Kieu et al. 2012). In maize, an adequate Fe nutritional status was found to reduce infection by *Colletotrichum graminicola* (Ye et al. 2014). These authors also reported that the beneficial role of Fe was dependent on the strength of Fe treatment (Ye et al. 2014). Very recently, iron redistribution was reported to induce oxidative burst and resistance against the fungal pathogen *Curvularia lunata* in maize (Fu et al. 2022). In wheat plants infected with *B. graminis* f.sp. *tritici*, Fe^3+^ accumulated at cell wall appositions to mediate an oxidative burst, intracellular iron depletion and induction of *PR* gene expression (Liu et al. 2007). From the information gained in maize, wheat and rice, it appears that Fe accumulation, and subsequent ROS production, might be a critical response to arrest pathogen infection in cereal species.

Although treatment with Fe has proven to be effective to protect rice plants from the rice blast fungus, it is anticipated that the iron status of a plant might have a different effect on disease resistance depending on the two partners, host plant and pathogen. Being a foliar pathogen, *M. oryzae* entirely depends on the host tissue to acquire essential nutrients required for pathogen growth, and both partners compete for Fe. In this multifaceted process, the plant can deploy strategies that restrict fungal invasion, either by sequestering Fe away from the pathogen (withholding strategy) or by accumulating Fe which can be toxic for the pathogen. On the other hand, pathogens might secrete iron-chelating siderophores to acquire Fe from their host (Herlihy et al. 2020). Further studies are required to elucidate the biological significance of the observed alterations in apoplastic Fe content in response to *M. oryzae* infection in rice plants. The pathogen lifestyle (necrotroph, biotroph, hemibiotroph) might also hold important implications to understand how Fe accumulation and distribution (intracellular and apoplastic Fe) might affect pathogen colonization in the host tissue. Then, it will be then of interest to investigate whether treatment with Fe confers resistance to pathogens with different lifestyles in rice.

## Conclusion

Results presented in this study demonstrated that, upon short-term exposure to Fe, rice plants develop a more robust defense response during infection with the rice blast fungus *M. oryzae* and confers blast resistance. Our results suggest that priming of plant defense by Fe treatment might find value for improving rice resistance against blast. The information gained in this study also illustrates the relevance of crosstalk between iron signaling and immune signaling pathways in controlling blast resistance in rice. A better understanding on the mechanisms underlying iron-immunity crosstalk will allow the development of appropriate strategies for protection of rice against the blast fungus.

## Materials and Methods

### Plant Growth Conditions

Rice (*O. sativa* cv. Nipponbare) plants were grown at 28 °C with a 14 h/10 h light/dark cycle. For Fe treatment, plants were grown in soil (turface:vermiculite:quartz sand [2:1:3]), and watered (bottom watering) with a half-strength Hoagland solution for 16 days (2.5 mM KNO_3_, 2.5 mM Ca(NO_3_)_2_·4H_2_O, 1 mM MgSO_4_·7H_2_O, 0.5 mM NH_4_NO_3_, 25 μM KH_2_PO_4_, 23.15 μM H_3_BO_3_, 4.55 μM MnCl_2_·4H_2_O, 0.38 μM ZnSO_4_·7H_2_O, 0.1 μM CuSO_4_·5H_2_O, 0.14 μM Na_2_MoO_4_·2H_2_O, 50 μM Fe-EDDHA, pH 5.5). To assess the effect of high Fe supply, the same nutrient solution was used but with a higher Fe concentration (1 mM Fe-EDDHA). The same procedure was used to examine the effect of low Fe treatment but using a nutrient solution containing 0.1 µM Fe-EDDHA. Treatments with the ferroptosis inhibitor, ferrostatin-1 (Fer-1) was carried out as previously described (Dangol et al. 2019).

### Blast Resistance Assays

The fungus *M. oryzae* (strain Guy11, courtesy of Ane Sesma) was grown in complete media agar (CMA) containing 30 µg/ml chloramphenicol) for 15 days at 28 °C under a 16 h/8 h light/dark photoperiod. *M. oryzae* spores were collected by adding sterile water to the surface of the mycelium and adjusted to the appropriate concentration.

Rice plants, control plants and Fe-treated plants (at 5 days of treatment), were spray-inoculated with *M. oryzae* spores (5 × 10^5^ spores/ml; 0.3 ml/plant) using an aerograph at 2 atm of pressure (see Fig. 1A, left panel, treatment with high-Fe plants; Additional File 1: Fig. S7, treatment with low-Fe) and maintained overnight in the dark under high humidity. At the time of fungal infection, control and iron-treated rice plants were at the 3–4 leaf stage. After inoculation with fungal spores, the plants were allowed to continue growth under the same Fe regime (control, high Fe, or low Fe), and photoperiod condition. The development of disease symptoms was followed over time. The percentage of leaf area affected by blast lesions was determined on the youngest developed leaf (third leaf) of the *M. oryzae*-infected plants using digital imaging software (APS Assess 2.0 program; n = 10) (Lamari 2008). Fungal biomass was quantified by quantitative PCR (qPCR) using specific primers for the *M. oryzae* 28S DNA gene (Qi and Yang 2002).

### Phenotypical Analysis and Chlorophyll Content

Shoot and root fresh weight (FW) was determined in three-week-old rice plants treated, or not, with high Fe (1 mM Fe) at 5 days or 19 days after the onset of treatment. At least 10 biological replicates were examined. Chlorophyll content was determined using the SPAD 502 Plus Chlorophyll Meter (Spectrum Technologies). SPAD readings were obtained in the youngest developed leaf of rice plants grown in different Fe concentrations (n = 10).

### Plant Tissue Staining

H_2_O_2_ determination was carried out using H_2_DCFDA (2′,7′-dichlorodihydrofluorescein diacetate) staining following the procedure described by Kaur et al. (2016). Briefly, the rice leaves were cut into pieces (3 cm) and incubated in a solution containing 10 μM H_2_DCFDA, vacuum infiltrated during 5 min, and then maintained in darkness at room temperature for 10 min. The samples were washed three times with distilled water and stored in 20% glycerol and immediately visualized. The fluorescence emitted by H_2_DCFDA was observed at 488 nm in an Leica DM6 microscope under GFP fluorescence (488 nm excitation, 509 nm emission). Fluorescence was quantified using ImageJ software.

For DAB staining, the rice leaves were cut in 3 cm pieces, incubated in a solution containing 1 mg/ml DAB, vacuum infiltrated during 30 min, and then maintained in the dark at room temperature overnight. Leaves were washed with 75% ethanol. Following DAB staining, the rice leaves were subjected to Perls staining.

Perls staining was conducted on leaves and leaf sheaths of High-Fe, Control Fe and Low-Fe rice plants that have been inoculated with *M. oryzae* spores (48 hpi) as described by Stacey et al. (2008) with some modifications. Briefly, rice leaves were vacuum-infiltrated in a fixing solution (chloroform:methanol:glacial acetic acid; 6:3:1, v/v) for 1 h and incubated overnight at room temperature. After washing with distilled water (three times), samples were vacuum infiltrated with a pre-warmed (37 °C) staining solution (4% HCl and 4% K-ferrocyanide at equal volumes) for 1 h, incubated 4 h more at 37 °C in the same solution without vacuum, and then washed three times with distilled water. The Perls reagent (potassium ferrocyanide), in the presence of reactive Fe^3+^, produces an insoluble blue precipitate (Prussian blue) (Roschzttardtz and Curie 2010). Sections were mounted in glycerol 50%. All the images were taken in an AixoPhot DP70 microscope under bright light. Three biological replicates with 4 technical replicates each were performed.

Trypan blue staining was performed to detect cell death in *M. oryzae*-infected rice leaves. For this, detached rice leaves were boiled in alcoholic lactophenol (96% ethanol-lactophenol (1:1 v/v) containing 0.1 mg/ml trypan blue for 8 min and cleared in 70% chloral hydrate solution overnight. Samples were stored in glycerol 70% until microscopic observation.

### Lipid Peroxidation Assay and BODIPY Staining

The level of lipid peroxidation was determined by measuring the amount of MDA produced by the thiobarbituric acid reaction as described by Campo et al. (2014). Briefly, samples (0.05 g) were homogenized in 1 ml of 80% (v/v) ethanol and centrifuged at 16,000*g* for 20 min at 4 °C. The supernatant (0.5 ml) was recovered and mixed with 0.5 ml of 20% (w/v) trichloroacetic acid containing 0.65% (w/v) thiobarbituric acid. The mixture was incubated at 85 °C for 30 min and then quickly cooled in an ice bath. After centrifugation (10,000*g*, 10 min), the absorbance of the supernatant was measured at 532 nm and 600 nm (Spectramax M3 reader, Molecular Devices, USA). The value for nonspecific absorption at 600 nm was substracted from the 532 nm reading. The MDA content was calculated using its molar extinction coefficient (156 mM^−1^ cm^−1^), and the results are expressed as mM MDA/g fresh weight (FW).

C11-BODIPY^581/591^ (Thermo Fisher Scientific) was used to visualize lipid peroxidation (10 µM C11-BODIPY^581/591^) following the procedure described by Shen et al. (2020). Epifluorescence of fluorescent dye was visualized using a Leica DM6 and the fluorescence emitted by oxidized C11-BODIPY^581/591^ was observed at 488 nm in an Leica DM6 microscope under GFP fluorescence (488 nm excitation, 509 nm emission).

### RNA Isolation, RNA-seq and Analysis of RNA-seq Data

Total RNAs were obtained using Maxwell(R) RSC Plant RNA Kit (Promega). For RNA-Seq, two biological replicates for each condition were examined, each biological replicate consisting of leaves from five individual plants (youngest developed leaf). RNA concentration and purity were checked using a spectrophotometer (NanoDrop, ND-1000). RNA quality and integrity was examined on an Agilent 2100 Bioanalyzer (Agilent Technologies, Inc.; RNA integrity number (RIN) ≥ 8). A total of eight libraries were subjected to RNA-Seq analysis (125 paired-end reads), yielding an average of 24,812,610 clean reads/library (Additional file 8: Table S7). Raw reads were processed and analyzed as previously described (Sánchez-Sanuy et al. 2019). Reads were mapped against the reference genome, *Oryza sativa* sp. *japonica* (IRGSP-1.0 Ensembl release 41). To identify differentially expressed genes, a FDR cutoff < 0.01 and log2FC − 0.5 ≤ or ≥ 0.5 was applied. Gene Ontology (GO) enrichment analysis (GOEA) was performed using the AgriGO web tool (Parametric Analysis of Gene Set Enrichment) (Du et al. 2010; https://bioinfo.cau.edu.cn/agriGOv2/). Enriched GO terms were clustered and plotted with the online analysis tool ReviGO (https://revigo.irb.hr/) (Supek et al. 2011).

Cluster analysis was used to determine the expression patterns of differentially expressed genes identified by RNA-Seq using the Panther gene ontology web tool (http://geneontology.org/). The FPKM value was used as the expression level. Heatmaps were drawn by R pheatmap package.

### Expression Analysis by qRT-PCR

Total RNA was extracted from plant tissues using TRizol reagent (Invitrogen). For quantitative RT-PCR (RT-qPCR), the first complementary DNA was synthesized from DNase-treated total RNA (0.5 μg) with High Capacity cDNA Reverse Transcription (Life technology, Applied Biosystems). Amplification involved cDNA (2 μl, 5 ng/μl) in optical 384-well plates (Roche Light Cycler 480; Roche Diagnostics, Mannheim, Germany) with SYBR Green I dye and gene-specific primers (Additional file 9: Table S8). The *Ubiquitin1* gene (Os06g0681400) was used to normalize transcript levels. Results from one of three independent experiments with similar results are shown. Four biological replicates which included RNA samples used for RNASeq analysis (two technical replicates each) were analyzed. Asterisks indicate significant differences calculated by two way ANOVA *, *P* ≤ 0.05; **, *P* ≤ 0.01; ***, *P* ≤ 0.001).

### Analysis of Total and Apoplastic Fe

For total Fe content, the youngest developed leaves (third leaf) were air-dried and then 25–50 mg were mineralized by a microwave digester system (MULTIWAVE-ECO, Anton Paar GmbH) in 65% (v/v) HNO_3_. Aliquots of the mineralized samples were adequately diluted in Milli-Q water and the concentrations of total Fe were measured by ICP-MS technique (Bruker Aurora M90 ICP-MS, Bruker Daltonik GmbH). In order to check the nebulization performance, an aliquot of 2 mg L^−1^ of an internal standard solution (^72^Ge, ^89^Y, and ^159^Tb) was added to the samples and calibration standards to give a final concentration of 20 µg L^−1^. Possible polyatomic interferences were removed by using CRI (Collision–Reaction-Interface) with an H_2_ flow of 80 mL/min flown though skimmer cone. Calibration curve were obtained using multi-ICP-MS standard solutions (Ultra Scientific, USA). Five biological replicates (three technical replicates each) were analyzed. Differences were calculated by one way ANOVA *, *P* ≤ 0.05; **, *P* ≤ 0.01).

The apoplast wash fluid (AWF) was obtained from the same plant material used for the analysis of total Fe content. For this, the youngest developed leaves of three-week-old plants (1 gr of fresh weight) were cut in 5 cm pieces and placed in a tube containing 30 ml of 200 mM CaCl_2_, 5 mM Na-acetate (pH 4.3 adjusted with glacial acetic acid) and 0.1 mM TPCK and 0.1 mM PMSF (freshly prepared), and kept on ice under constant agitation for 1 h. Then, vacuum was applied for 30 min and leaves were removed and carefully dried. Dried leaves were rolled and placed in a tube for AWF recovery. Leaves were centrifuged a 3000 rpm, 15 min at 4 °C. AWF was recovered from the bottom of the tube and kept in − 20 until use.

To assess that the apoplast fluid was devoid of cytoplasmic contaminants we assayed malate dehydrogenase (MDH) activity (Goulet et al. 2010). For each material, MDH activity in apoplast samples was compared with that of total protein extracts. Total protein extracts were prepared from rice leaves using 1 mM MOPS, pH 7.5, containing 5 mM NaCl, and protease inhibitors (TPCK and PMSF, 0.1 mM each). Protein concentrations of total and apoplastic fluid extracts was quantified using Bradford. For MDH assay, protein samples (10 μl of either total protein or apoplastic fluid extracts, 0.1 μg/μl) were added to the reaction mixture containing 0.4 mM NADH, 0.2 mM oxaloacetic acid, 1 mM MOPS, pH 7.5, in 96 micro titer plates. The absorbance at 340 nm at 25 °C was recorded during 10 min. MDH activity was calculated as (Δabsorbance 340 nm/min/mg). Malate Dehydrogenase (MDH) activity and was found to be in the range of 3.5–4% compared to total leaf protein extracts.

Apoplastic Fe concentrations weredetermined by ICP-MS technique (see above) after diluting 30 µL of the apoplastic solutions to 1.2 mL with 2% HNO_3_ in (v/v) bidistilled water. Calibration curve were obtained using multi-ICP-MS standard solutions (Ultra Scientific, USA). Five biological replicates (three technical replicates each) were analyzed. Differences were calculated by one way ANOVA *, *P* ≤ 0.05; **, *P* ≤ 0.01).

### Quantification of Ferrous (Fe^2+^) and Ferric Fe (Fe^3+^)

For quantification of Fe^2+^ and Fe^3+^, we used the ferrozine assay described by Adolfsson et al. (2017) with some modifications. Specifically, dried leaf samples (50–70 mg of the youngest developed leaves of three-week-old plants, 5 plants each condition) were pulverized in a Tissue Lyser and incubated in 1 M HCl (700 μl) overnight at room temperature under gentle shaking. After centrifugation (10,000 rpm, 5 min, room temperature), the supernatant was recovered. For determination of Fe^2+^, 15 μl of 2 mM Ferrozine in 50 mM ammonium acetate pH 9.5 were added to 150 μl of plant extract. Fe^3+^ was determined after reduction to Fe^2+^ with 50% (v/v) hydroxylamine hydrochloride 6.25 M (a reducing agent that converts Fe^3+^ to Fe^2+^) added to 150 μl of the samples. After 20 min at room temperature, 15 μl of 2 mM Ferrozine were added to the samples. Ferrozine, in the presence of ferrous ions gives a pink-purple color which can be measured spectrophotometrically. For each sample, the background was determined in the ammonium acetate buffer and subtracted from values obtained in the corresponding Ferrozine-treated samples. Equivalent volumes of 1 M HCl and hydroxylamine hydrochloride (in 1 M HCl) were used as blanks for non-reduced and reduced samples, respectively. Absorbance was read at 562 nm. Fe^3+^ concentration was estimated by subtracting reduced hydroxylamine values with Fe^2+^ values. Standard curves using increasing Fe concentrations were performed. Five biological replicates with two technical replicates each were examined Asterisks indicate significant differences calculated by two-way ANOVA *, *P* ≤ 0.05; **, *P* ≤ 0.01).

### Quantification of Rice Phytoalexins

For quantification of rice phytoalexins, leaf segments (aprox. 2 cm in length, 200–300 mg) were mixed with 40 vol. of 70% methanol and incubated at 4 °C overnight with constant rotation. A 1 ml aliquot was centrifuged at maximum speed to remove cell debris and used for quantification of phytoalexins. Phytoalexins were quantified by liquid chromatography-tandem mass spectrometry (LC–MS/MS) as previously described (Miyamoto et al. 2016). Three biological replicates with two technical replicates each were performed. Significant differences in phytoalexin accumulation were evaluated with two-way ANOVA *, *P* ≤ 0.05; **, *P* ≤ 0.01).

### Supplementary Information

**Additional file 1: Fig. S1.** Characterization of rice plants grown under different Fe conditions. **Fig. S2.** Venn diagrams representing transcriptional changes in response to *M. oryzae* infection in leaves of High-Fe and Control plants. **Fig. S3.** GO enrichment analysis of genes down-regulated by *M. oryzae* infection in Control and High-Fe plants (48 hpi). **Fig. S4.** Hierarchical clustering of differentially expressed genes by RNA-Seq analysis. **Fig. S5.** Phenylpropanoid biosynthesis pathway. Genes whose expression is regulated by *M. oryzae* infection are indicated. **Fig. S6.** Expression of diterpene phytoalexin biosynthetic genes in leaves of Control and High-Fe plants (−, mock-inoculated; +, *M. oryzae*- inoculated). **Fig. S7**. Accumulation of phytoalexins in leaves of Control and Low-Fe plants. **Fig. S8.** Expression of genes involved in Fe homeostasis in leaves of Control and High-Fe plants (−, mock-inoculated; +, *M. oryzae*- inoculated). **Fig. S9.** Malate dehydrogenase (MDH) assay in total protein extracts and apoplast fluid from Control and High-Fe plants (mock-inoculated and *M. oryzae*- inoculated). **Fig. S10.** Trypan blue staining for cell death in *M. oryzae*-infected leaves of Control and High-Fe rice plants. **Fig. S11**. Accumulation of Fe in sheaths of Low-Fe, Control-Fe, and High-Fe in rice plants that have been treated with the ferroptosis inhibitor Ferrostatin-1 (+ Fer-1), or not (− Fer-1).

**Additional file 2: Table S1.** Differentially expressed genes (DEGs) in leaves of High-Fe plants relative to Control plants identified by RNASeq analysis (no infection conditions).

**Additional file 3: Table S2.** List of differentially expressed genes (DEGs) in leaves of High-Fe (**S2a**) and Control (**S2b**) plants at 48 hpi with *M. oryzae* spores, relative to the corresponding mock-inoculated plants.

**Additional file 4: Table S3.** GO enrichment analysis of genes regulated by *M.oryzae* infection in High-Fe and Control plants relative to the corresponding mock-inoculated plants.

**Additional file 5: Table S4.** Expression data of defense-related genes in leaves of Control and High-Fe plants during *M. oryzae* infection (48 hpi).

**Additional file 6: Table S5**. Expression data (FPKM and Log_2_FC) of genes involved in the biosynthesis of diterpene phytoalexins and sakuranetin in leaves of Control and High-Fe plants (*M. oryzae*-infected and mock-inoculated plants).

**Additional file 7: Table S6.** Expression data (FPKM and Log_2_FC) of genes involved in Fe homeostasis in leaves of Control and High-Fe plants (*M. oryzae*-infected and mock-inoculated plants).

**Additional file 8: Table S7.** Statistics for RNAseq analysis of leaves from Control and High-Fe plants, mock-inoculated and *M. oryzae*-inoculated (48 hpi).

**Additional file 9: Table S8.** List of oligonucleotides used in this study.

## Data Availability

The RNA sequence datasets generated in this study can be found at the National Center for Biotechnology Information (NCBI) Gene Expression Omnibus (GEO) with the accession number “GSE202997” (https://www.ncbi.nlm.nih.gov/geo/query/acc.cgi?acc=GSE202997).
