# Supplementary material for: Iron Induces Resistance Against the Rice Blast Fungus Magnaporthe oryzae Through Potentiation of Immune Responses
Source: Rice (N Y). 2022 Dec 25;15:68. doi: 10.1186/s12284-022-00609-w (PMC9790844; doi:10.1186/s12284-022-00609-w)
Supplement: Supplementary file 1 — Additional file 1: Fig. S1. Characterization of rice plants grown under different Fe conditions. Fig. S2. Venn diagrams representing transcriptional changes in response to M. oryzae infection in leaves of High-Fe and Control plants. Fig. S3. GO enrichment analysis of genes down-regulated by M. oryzae infection in Control and High-Fe plants (48 hpi). Fig. S4. Hierarchical clustering of differentially expressed genes by RNA-Seq analysis. Fig. S5. Phenylpropanoid biosynthesis pathway. Genes whose expression is regulated by M. oryzae infection are indicated. Fig. S6. Expression of diterpene phytoalexin biosynthetic genes in leaves of Control and High-Fe plants (−, mock-inoculated; +, M. oryzae- inoculated). Fig. S7. Accumulation of phytoalexins in leaves of Control and Low-Fe plants. Fig. S8. Expression of genes involved in Fe homeostasis in leaves of Control and High-Fe plants (−, mock-inoculated; +, M. oryzae- inoculated). Fig. S9. Malate dehydrogenase (MDH) assay in total protein extracts and apoplast fluid from Control and High-Fe plants (mock-inoculated and M. oryzae- inoculated). Fig. S10. Trypan blue staining for cell death in M. oryzae-infected leaves of Control and High-Fe rice plants. Fig. S11. Accumulation of Fe in sheaths of Low-Fe, Control-Fe, and High-Fe in rice plants that have been treated with the ferroptosis inhibitor Ferrostatin-1 (+ Fer-1), or not (− Fer-1). [file 12284_2022_609_MOESM1_ESM.pdf]

Figure S1. Sánchez Sanuy

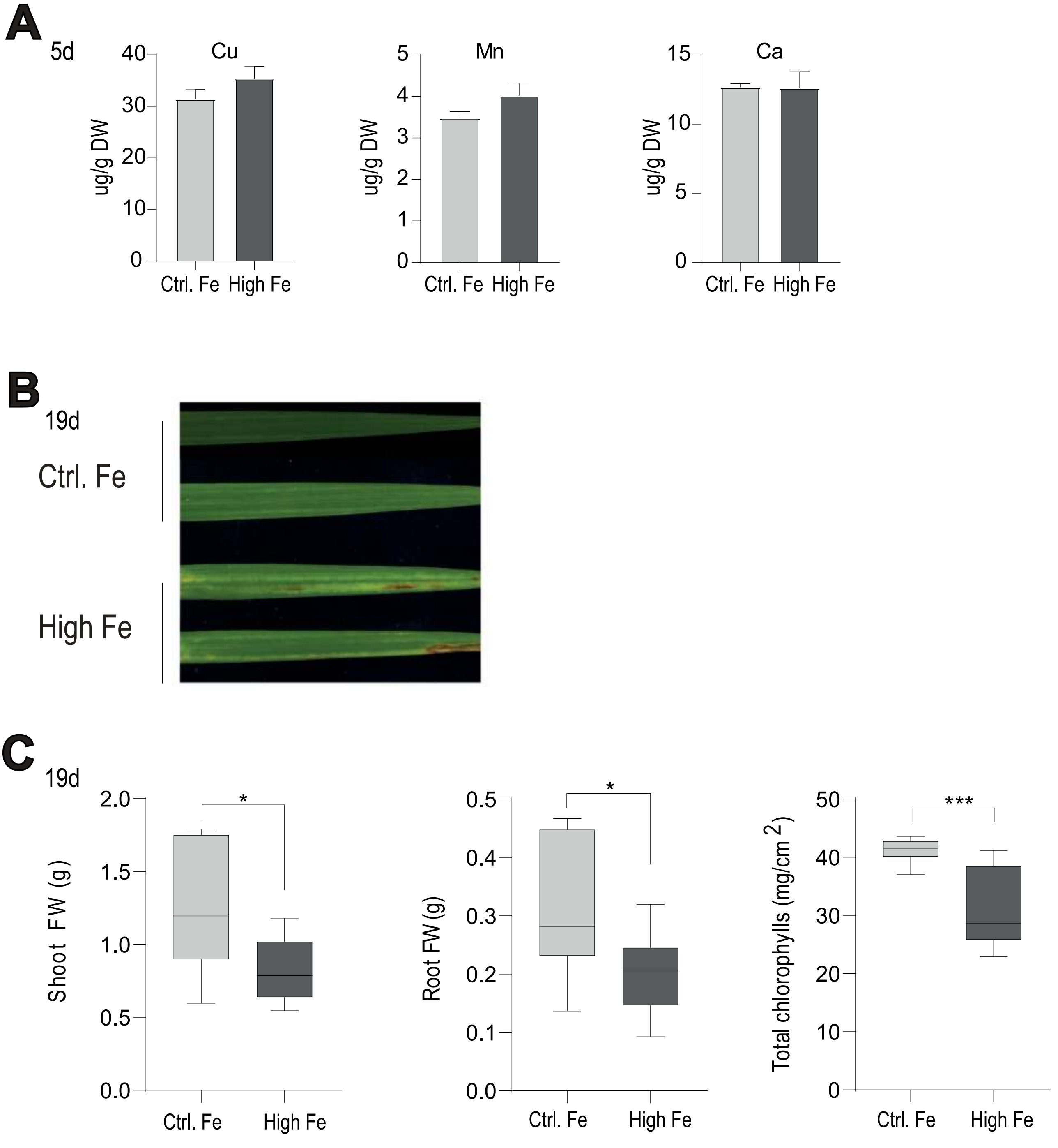

Supplemental Figure S1. Characterization of rice plants grown under different Fe conditions. Plants were grown in soil under control Fe conditions for 16 days and then allowed to continue growth for 5 days (A) or 19 days (B, C) more under high Fe (1 mM Fe, High-Fe) or control conditions (50 µM Fe, Ctrl. Fe). (A) Cu, Mn and Ca content in leaves of Control and High Fe plants after 5 days of treatment with high Fe as determined by ICP-MS. Five independent biological replicates (three technical replicates each) were analyzed. No statistical significance was observed between Control and High Fe plants (t-test). (B) Leaves of Control and High Fe plants after 19 days of treatment with high Fe. (C) Shoot and root fresh weight (FW) in Control and High Fe plats after 19 days of treatment with high Fe (left and middle panels), and chlorophyll content (right panel). Data are mean ± SEM (n=10). Asterisks denote statistically significant differences (t-test, *, $P < 0.05$, ***, $P < 0.001$).

Figure S2. Sánchez Sanuy

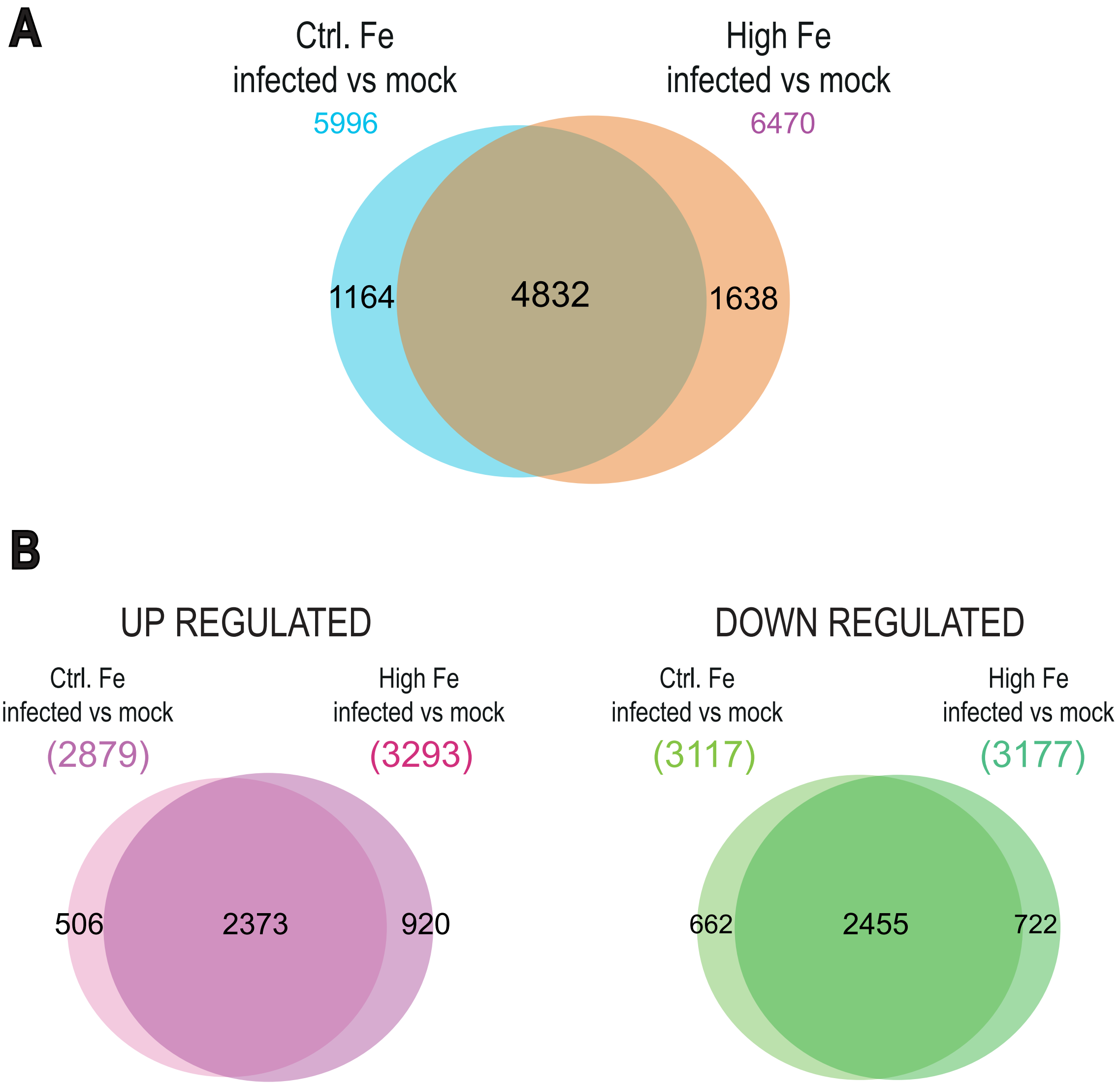

Supplemental Figure S2. Venn diagrams representing transcriptional changes in response to *M. oryzae* infection in leaves of High-Fe and Control plants. **(A)** Number of genes that are specifically and commonly regulated by *M. oryzae* infection in each Fe condition (Control and High Fe) at 48 hpi. **(B)** Genes that are up-regulated (purple, left panel) or down-regulated (green, right panel) in each Fe condition (log_2 fold change >0.5 or <-0.5; P-value ≤ 0.05).

Figure S3. Sánchez Sanuy

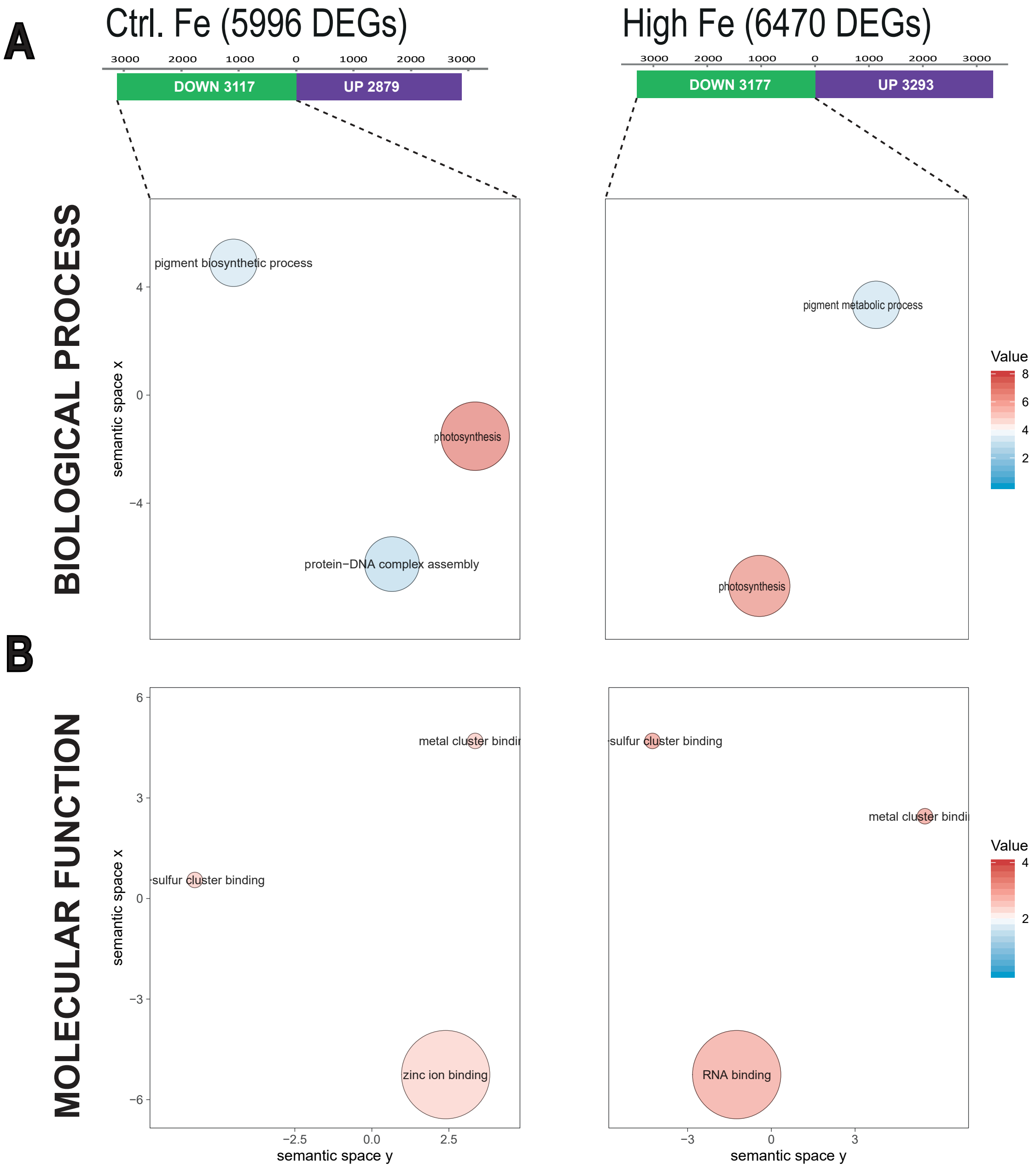

Supplemental Figure S3 GO enrichment analysis of genes down-regulated by *M. oryzae* infection in Control and High-Fe plants (48 hpi) in the categories of **(A)** Biological Processes and **(B)** Molecular Function. GO terms were visualized using REVIGO (https://revigo.irb.hr/) after reducing redundancy and clustering of similar GO terms in the *O. sativa* database. GO terms are represented by circles and are clustered according to semantic similarities (more general terms are represented by larger size circles, and adjoining circles are most closely related). Circle size is proportional to the frequency of the GO term, whereas color indicates the enrichment derived from the AgriGO analysis (red higher, blue lower). Full data sets of DEGs and lists of complete GO terms are presented in Supplemental Table S2 and S3, respectively.

Figure 4. Sánchez Sanuy

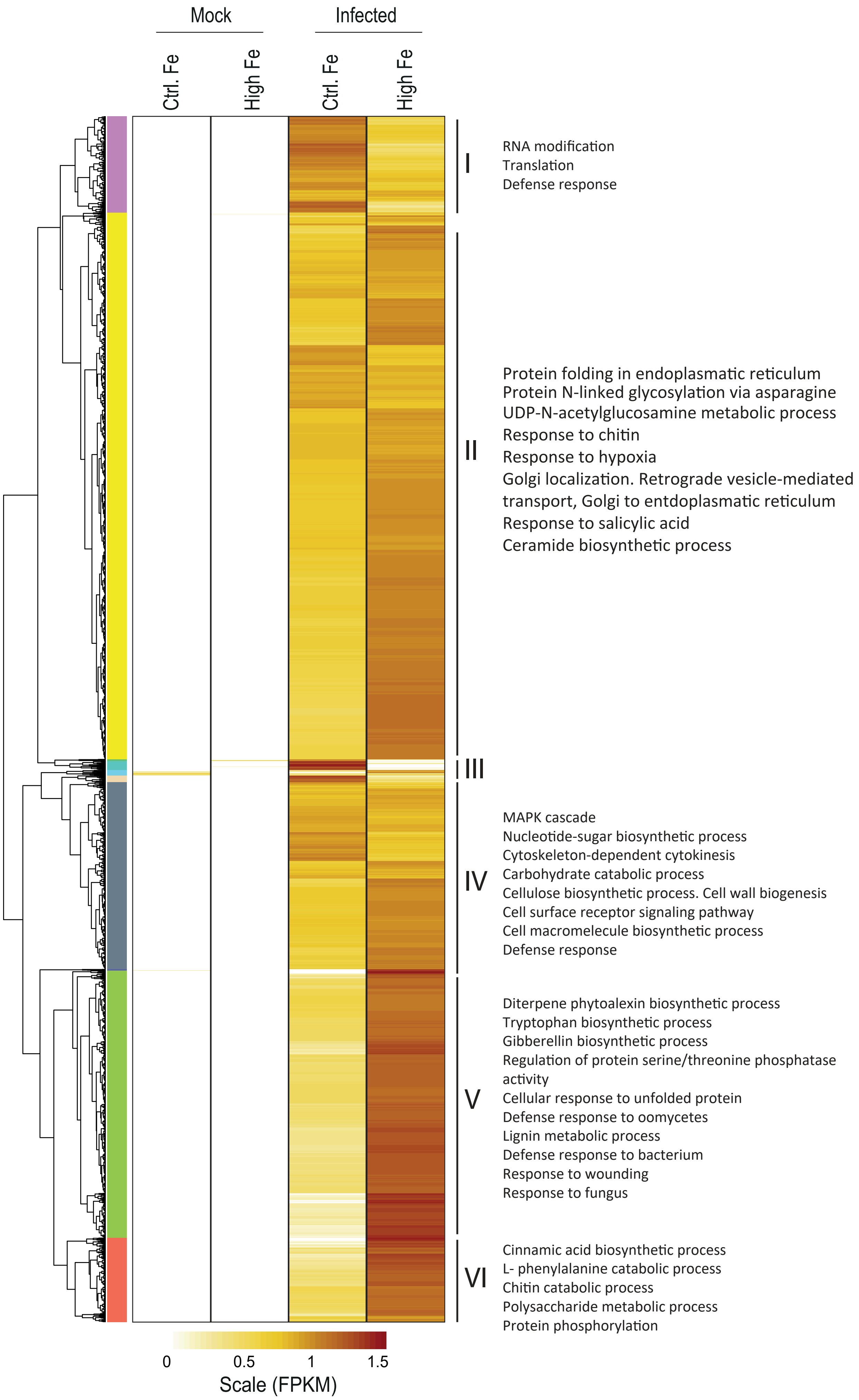

Figure S5. Sánchez Sanuy

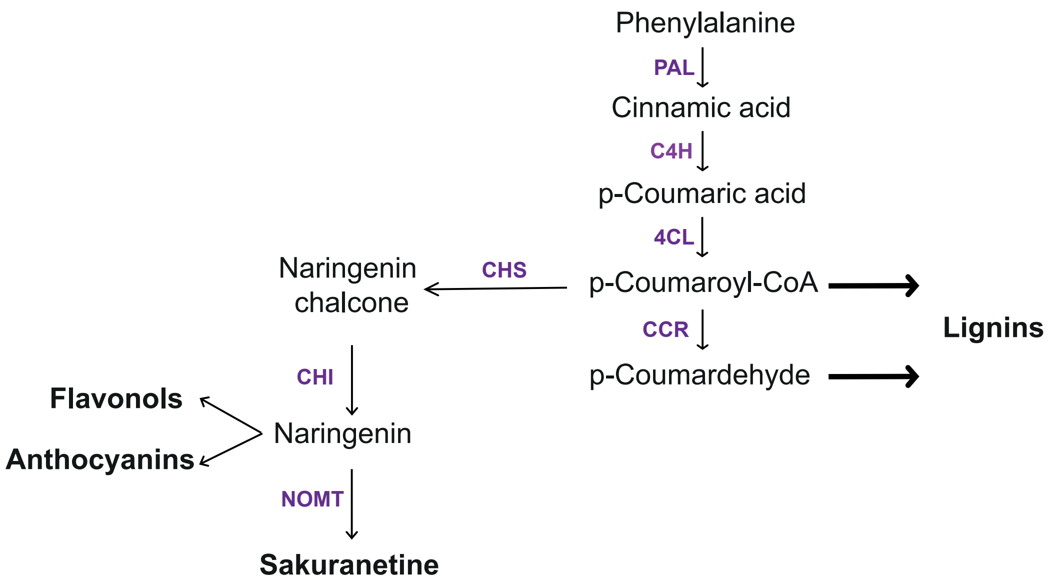

Supplemental Figure S5. Phenylpropanoid biosynthesis pathway. Genes whose expression is regulated by *M. oryzae* infection are indicated in purple. **PAL**, phenylalanine ammonia lyase; **C4H**, cinnamate-4-hydroxylase; **4CL**, 4-coumaroyl-CoA ligase; **CCR**, cinnamoyl-CoA reductase; **CHS**, chalcone synthase; **CHI**, chalcone isomerase; **NOMT**, naringenin 7-O-methyltransferase.

Figure S6. Sánchez Sanuy

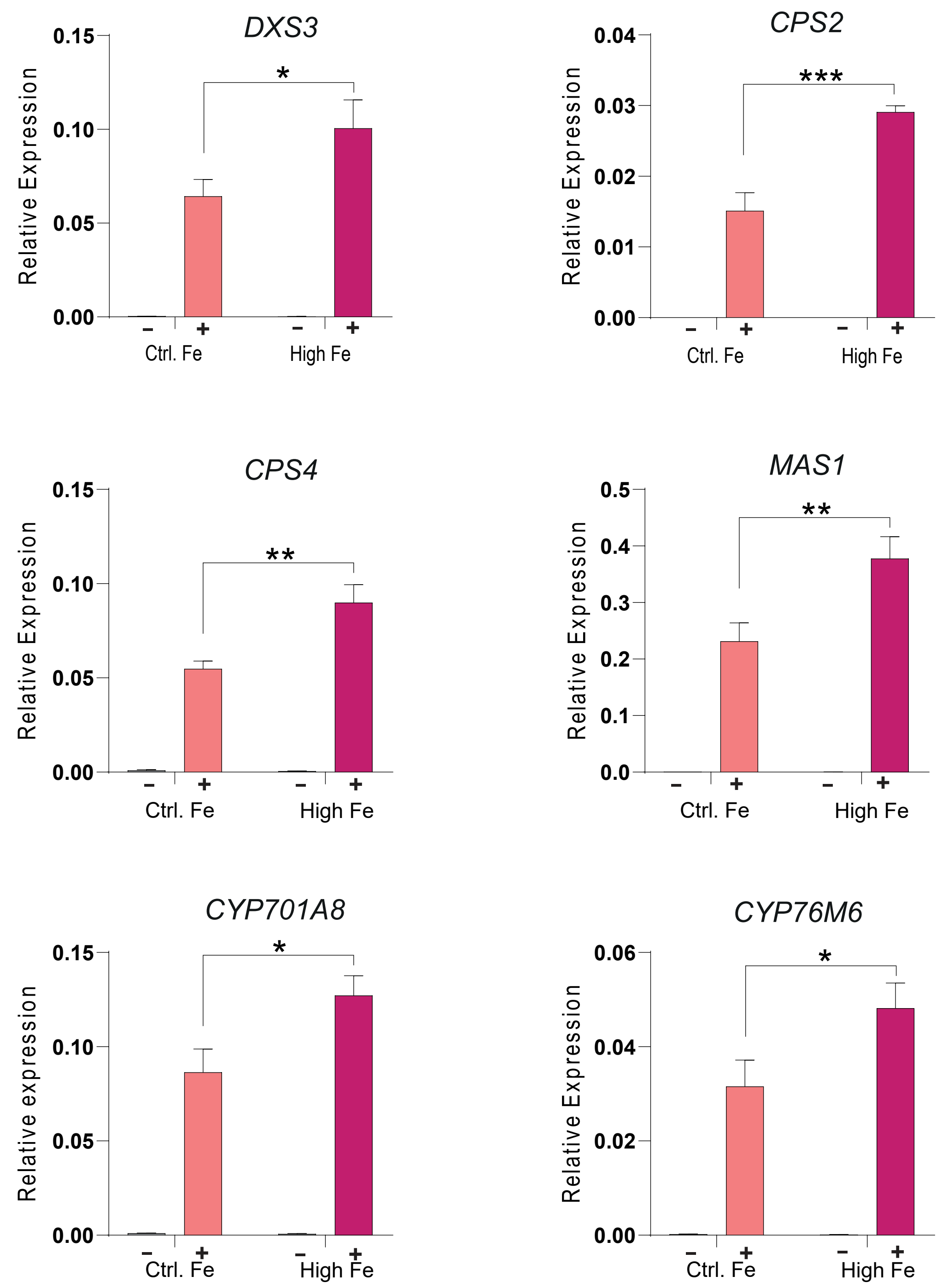

Supplemental Figure S6. Expression of diterpene phytoalexin biosynthetic genes in leaves of Control and High-Fe plants (-, mock-inoculated; +, *M. oryzae*- inoculated). Transcript levels were determined by RT-qPCR analysis at 48 hpi. The expression values were normalized to the rice Ubiquitin1 gene. Four independent biological replicates (2 technical replicates each) were assayed. Data are mean ± SEM (n=3). Asterisks indicate statistical significant differences calculated by two-way ANOVA (*, **, and *** indicate $P < 0.05$, 0.01, and 0.001, respectively). Gene-specific primers are listed in Supplemental Table S8.

Figure S7. Sánchez Sanuy

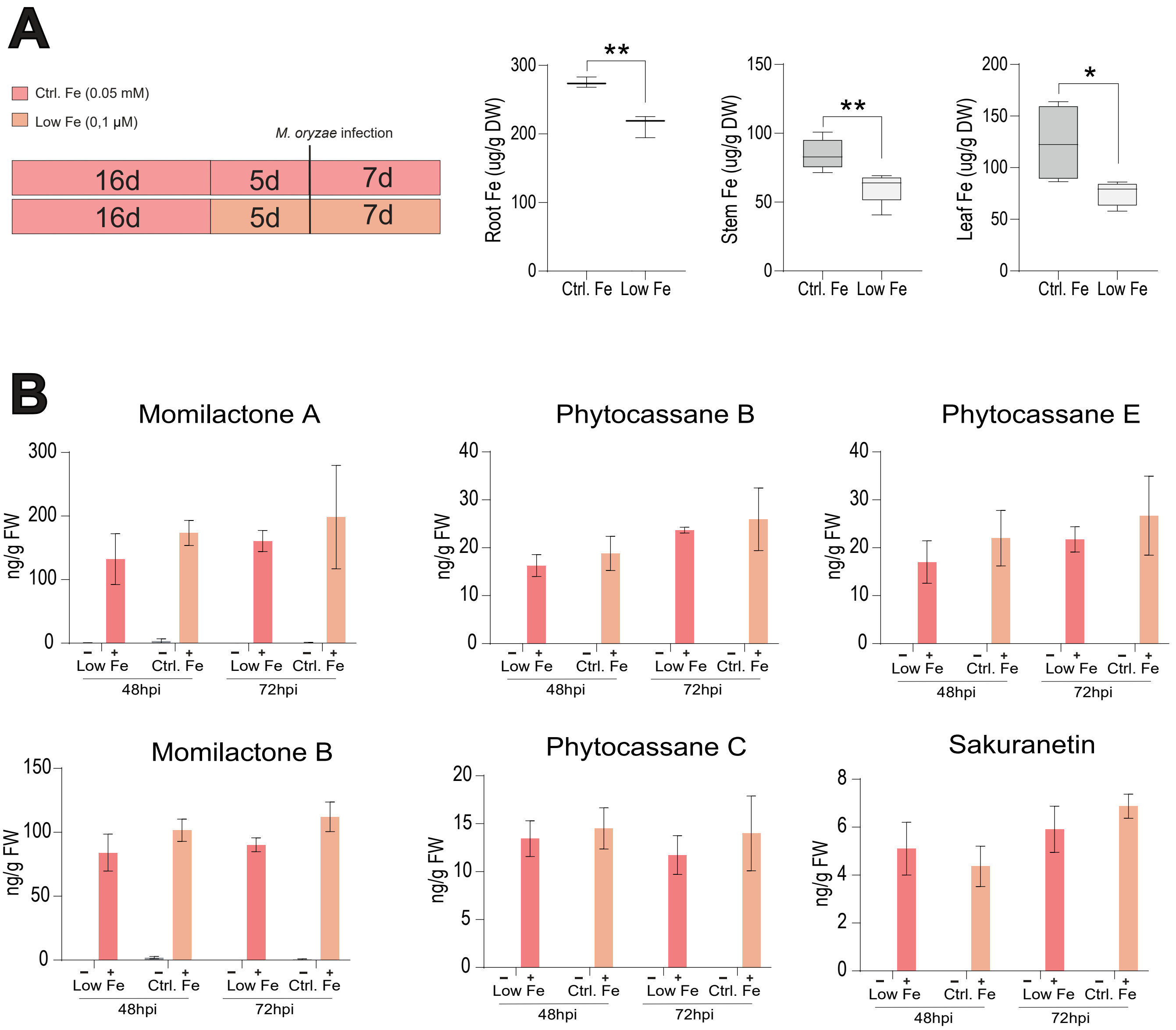

Supplemental Figure S7. Accumulation of phytoalexins in leaves of Control and Low-Fe plants. A. Experimental design used for treatment with low Fe and infection with *M. oryzae* ($5x10^5$ spores/ml) (left panel). Right panel, total Fe content estimated by the ferrozine colorimetric method in roots, stems and leaves of Control and Low-Fe-treated plants. Data are mean ± SEM (n = 10). Asterisks indicate statistical significant differences (t-test, *, $P < 0.05$, **, $P < 0.01$). B. Accumulation of phytoalexins, diterpenoid phytoalexins, momilactones (A and B), phytocassanes (B, C and E), and the flavonoid phytoalexin sakuranetin at 48 hpi and 72 hpi with *M. oryzae* spores (-, mock-inoculated; +, *M. oryzae*-inoculated). Data are mean ± SEM of three biological replicates each with 5 plants. There were no statistically significant differences among *M. oryzae*-infected plants (two-way ANOVA).

Figure S8. Sánchez Sanuy

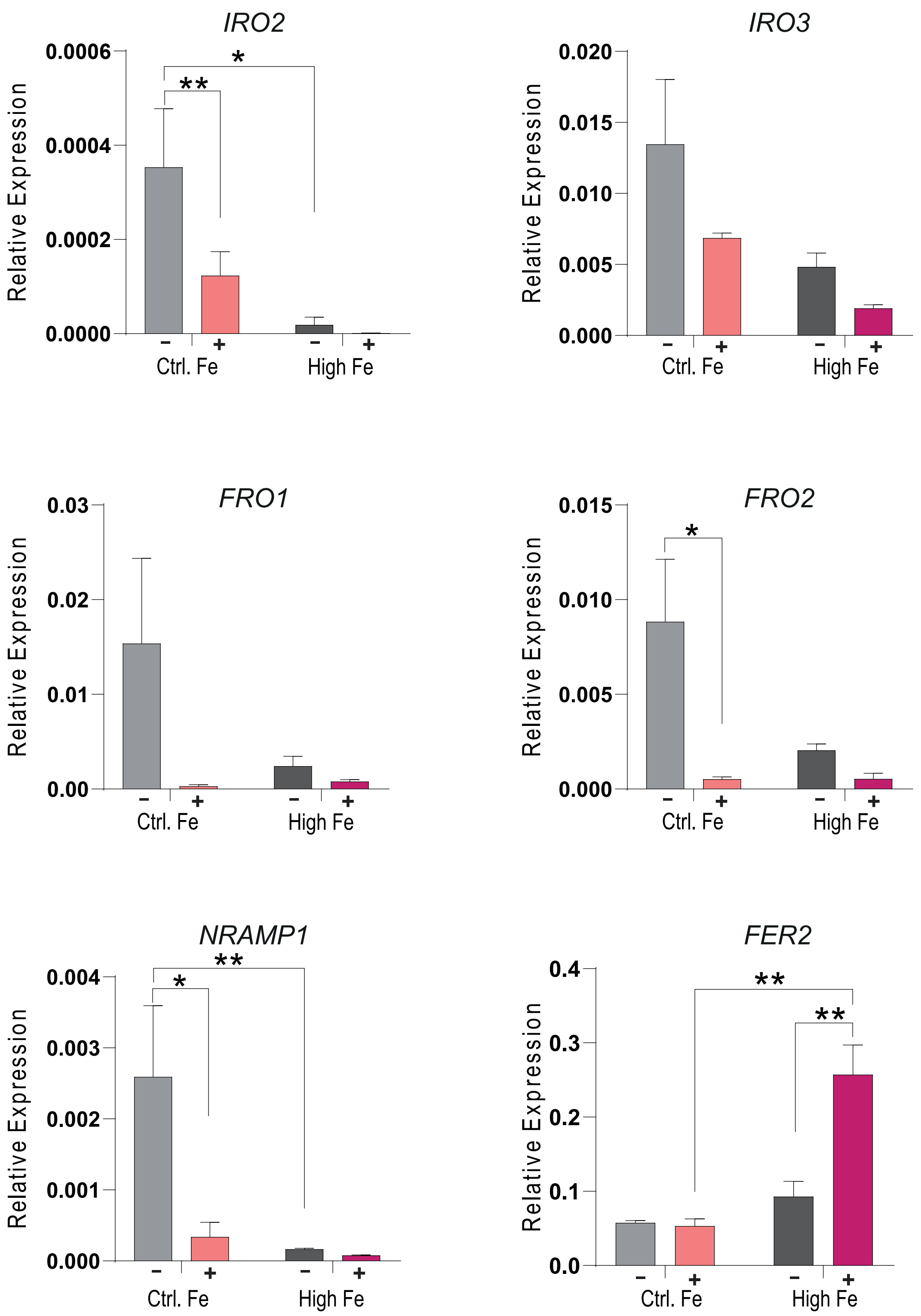

Supplemental Figure S8. Expression of genes involved in Fe homeostasis in leaves of Control and High-Fe plants (-, mock-inoculated; +, *M. oryzae*- inoculated). Transcript levels were determined by RT-qPCR analysis at 48 hpi. The expression values were normalized to the rice Ubiquitin1 gene. Four independent biological replicates (2 technical replicates each) were assayed. Data are mean ± SEM (n=3). Asterisks indicate statistical significant differences calculated by two-way ANOVA (*, $P < 0.05$, **, $P < 0.01$). Gene-specific primers are listed in Supplemental Table S8.

Figure S9. Sánchez Sanuy

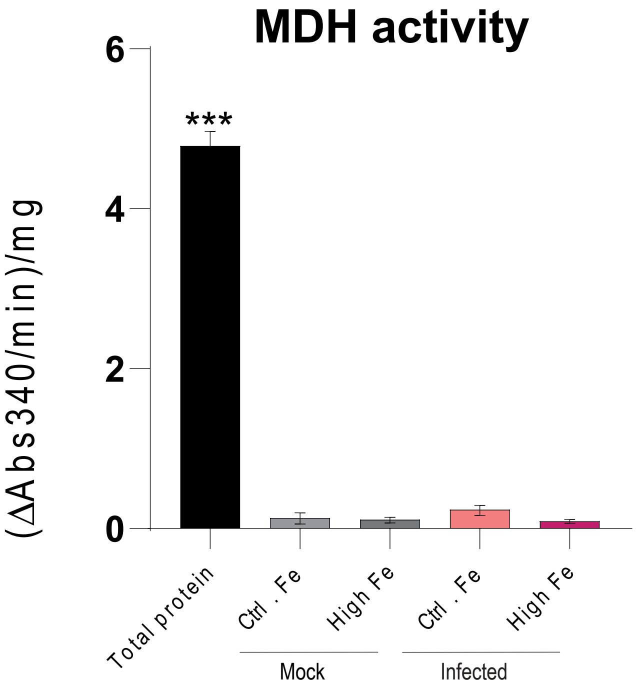

Supplemental Figure S9. Malate dehydrogenase (MDH) assay in total protein extracts (black bar) and apoplast fluid obtained from Control and High-Fe plants (mock-inoculated and *M. oryzae*- inoculated). Five biological replicates for each condition (each consisting in a pool of 10 leaves from individual plants) were analyzed. Asterisks indicate statistical significant differences calculated by two-way ANOVA (*** P ≤ 0.001).

Figure S10. Sánchez Sanuy

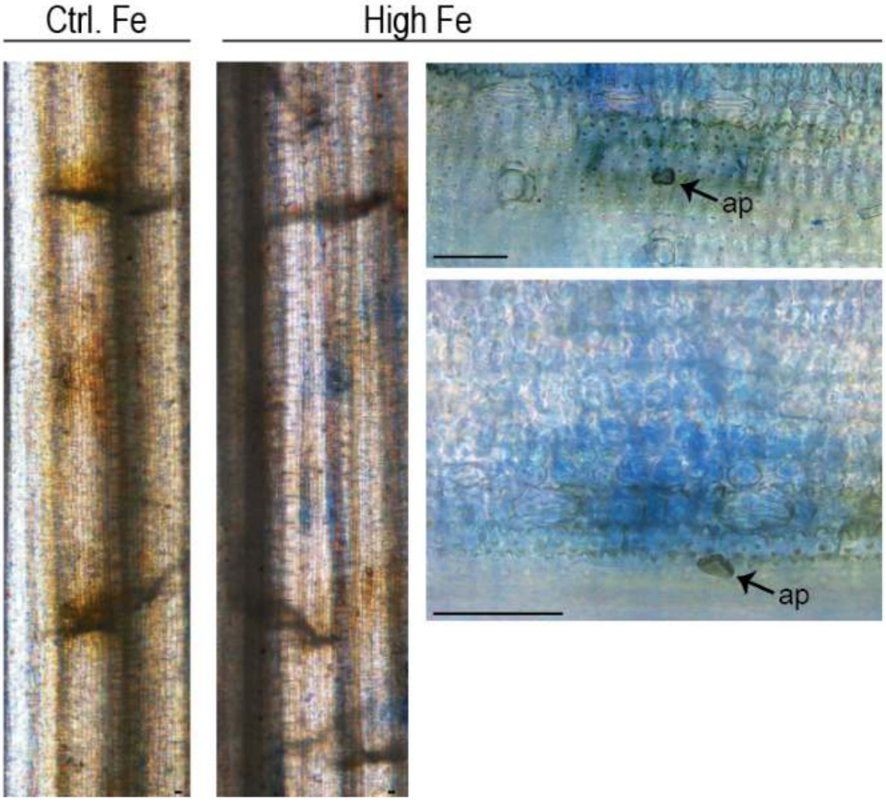

Supplemental Figure S10. Trypan blue staining for cell death in *M. oryzae*-infected leaves of Control and High-Fe rice plants (at 24 hpi). Bars correspond to 50 μm. Ap, appresorium.

Figure S11. Sánchez Sanuy

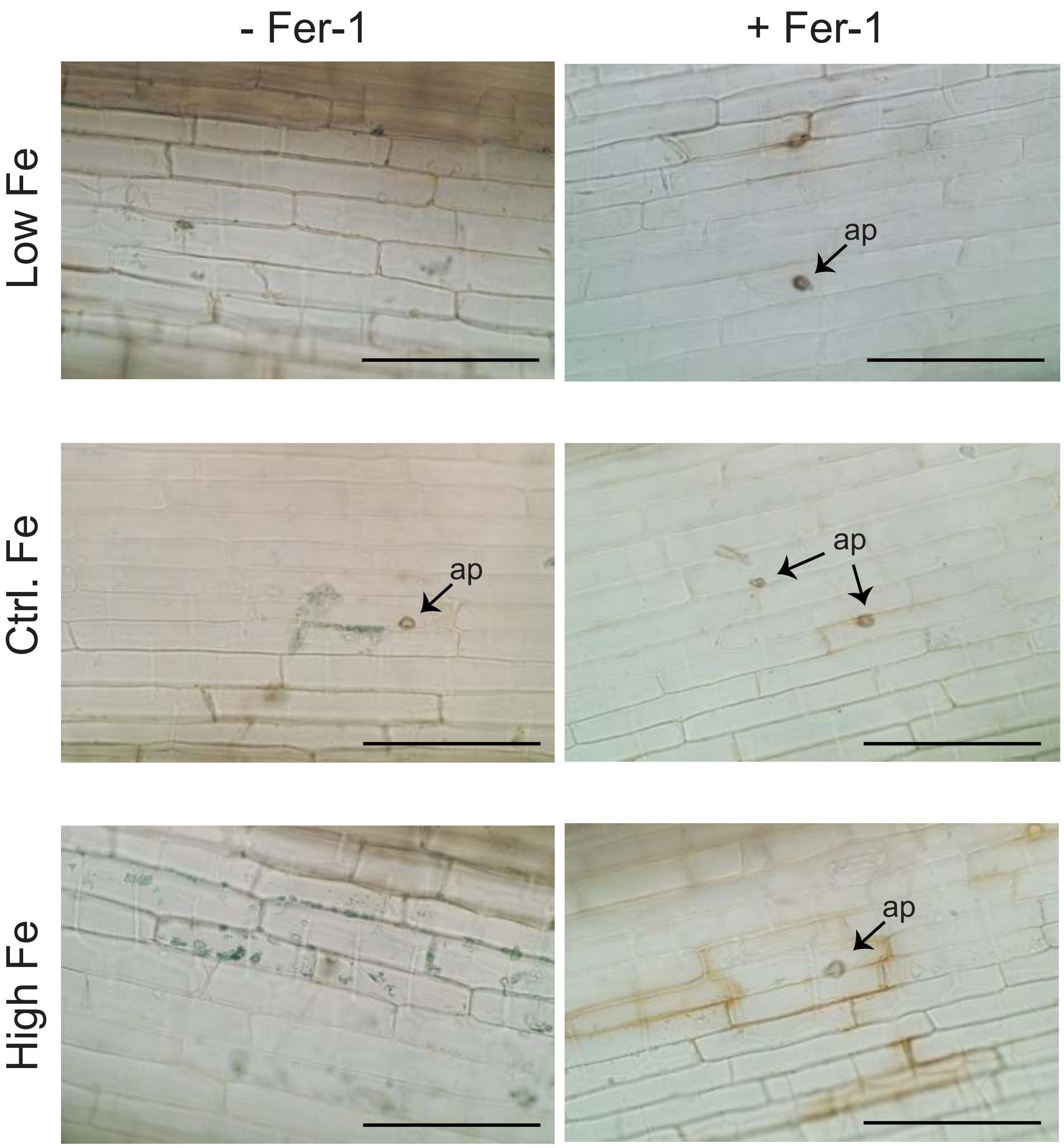

Supplemental Figure S11. Accumulation of Fe in sheaths of Low-Fe, Control-Fe, and High-Fe rice plants that have been treated with the ferroptosis inhibitor Ferrostatin-1 (+ Fer-1), or not (- Fer-1). Bars correspond to 100 μm. ap, appresorium.
